# Enhancing whole genome DNA amplification of *Plasmodium falciparum* for advanced molecular surveillance in malaria control

**DOI:** 10.1186/s12936-025-05643-9

**Published:** 2025-12-19

**Authors:** Mark K. I. Tan, Myrela C. S. de Jesus, Jody E. Phelan, Joseph Thorpe, Daniel Ward, Rahul Batra, Amita Patel, Debbie Nolder, Peter Chiodini, Colin J. Sutherland, Jonathan Edgeworth, Taane G. Clark, Susana Campino

**Affiliations:** 1 Department of Infectious Diseases, King’s College London, London, SE1 1UL UK; 2 Centre for Clinical Infection and Diagnostics Research, Guy’s & St. Thomas’ NHS Foundation Trust, London, SE1 7EH UK; 3 Faculty of Infectious and Tropical Diseases, London School of Hygiene and Tropical Medicine, London, WC1E 7HT UK; 4 Department of Parasitology, Institute of Biomedical Sciences, University of São Paulo, São Paulo, Brazil; 5 Malaria Reference Laboratory, UK Health Security Agency, London School of Hygiene and Tropical Medicine, Keppel Street, London, WC1E 7HT UK; 6 Faculty of Epidemiology and Population Health, London School of Hygiene and Tropical Medicine, London, WC1E 7HT UK

**Keywords:** Malaria, *Plasmodium falciparum*, Whole genome sequencing, Selective whole genome amplification, Nanopore sequencing, Optimisation

## Abstract

**Background:**

Genomics is critical for malaria control and elimination by providing population-level monitoring of malarial gene flow. Whole genome sequencing is valuable for identifying such genomic changes and antimalarial drug resistance. However, sequencing submicroscopic and asymptomatic cases presents challenges due to their low parasite densities and relative abundance of human DNA. Selective whole genome amplification (SWGA) is a method that employs oligonucleotide primers and *phi29* DNA polymerase to specifically amplify *Plasmodium* DNA. SWGA has proven effective in generating increased genome coverage for various *Plasmodium* species. Despite its promise, current SWGA protocols have proven unsuitable in routine clinical settings. This study optimizes the SWGA protocol to improve amplification efficiency and simplify processing of clinical samples.

**Methods:**

Thirteen *P. falciparum* clinical whole blood samples from returning travellers underwent SWGA with varying incubation durations of 2, 8, and 16 h. Using a newly developed SWGA protocol, three samples were additionally incubated at 3 h with EquiPhi29™ DNA Polymerase, a newer version of the *phi29* enzyme. Effectiveness of the varying conditions were compared by their amplification and sequencing yield, parasite DNA recovery, genome coverage and coverage depth. After read number normalization through random selection with *rasusa*, pairwise SNP comparison was also performed to ensure variant calling by *freebayes* was unaffected by the changes in condition. Pairwise agreement was tested by Cohen’s Kappa. Drug resistance profiles for each sample were generated with *Malaria-Profiler* to further verify these findings.

**Results:**

The results demonstrate that there were comparable amplification and sequencing yields between the 8 and 16 h SWGA durations. These durations also showed significant results similarities in variant calling with up to > 90% SNP concordance. Moreover, using the newer developed SWGA protocol, sequencing yields and genome coverage were significantly enhanced, achieving over 63% *P. falciparum* genome coverage in just three hours processing prior to sequencing. While reducing reagent costs by almost 60%. Overall, drug resistance profiles remained consistent across all tested conditions.

**Conclusion:**

This advance holds promise for faster, more cost-effective malaria diagnostics and genomic surveillance, improving clinical decision-making and supporting malaria elimination efforts.

**Supplementary Information:**

The online version contains supplementary material available at 10.1186/s12936-025-05643-9.

## Background

Malaria, caused by *Plasmodium* spp. parasites, has a high global impact, with an estimated 247 million cases and 619,000 deaths in 2023 alone [1]. The World Health Organization (WHO) Global Technical Strategy for Malaria aims for a 90% reduction in the disease burden by 2030, including elimination across 35 countries [2]. However, progress towards malaria elimination is threatened by the emergence and spread of antimalarial drug resistance, and this is particularly important for *Plasmodium falciparum*. Variant alleles of several *P. falciparum* genes have been associated with antimalarial drug resistance, including *pfcrt* and *pfmdr1* for chloroquine and amodiaquine, *pfdhps* and *pfdhfr* for sulfadoxine-pyrimethamine (SP), and *pfkelch13* for artemisinin, compromising the efficacy of current frontline artemisinin-based combination therapy (ACT) [3]. With the establishment of ACT-resistance across the Mekong region and novel emergence in Africa [4–7], genomics-based methods are not only increasingly important for surveillance but also to provide the correct treatment through a rapid diagnostic approach [8]. However, sequencing parasites from infected individuals, who often present with low parasitaemia, remains challenging but is critical for infection control and elimination efforts.

Selective whole genome amplification (SWGA) is a pathogen enrichment method used to enhance *Plasmodium* parasite DNA for whole genome sequencing (WGS). It has been recommended for use in samples provided by both symptomatic and asymptomatic individuals, including field-collected faecal and blood samples from those with low parasitaemia [9, 10]. SWGA utilizes sets of oligonucleotide primers, which are designed to bind preferentially and at higher frequency to the target *Plasmodium* DNA compared to the human DNA present in a sample [11]. When combined with high fidelity *phi29* DNA polymerases and multiple displacement amplification (MDA), large targeted DNA segments can be amplified [12, 13]. Specifically, SWGA overcomes the main challenge for *Plasmodium* WGS from low parasite density material, which is the high relative abundance of host DNA from circulating leukocytes; thereby, achieving feasibility for the sequencing of parasite genomes from infected individuals while negating the need for leukocyte filtering. Several SWGA strategies have been developed to support WGS of *P. falciparum*, *Plasmodium vivax*, *Plasmodium knowlesi*, *Plasmodium malariae*, and *Plasmodium ovale* spp. parasites from clinical samples [14, 15]. The SWGA-amplified DNA was found to improve WGS efficiency and the resulting genome coverage and sequencing depth [16], providing greater accuracy and SNP detection [10].

Alongside improving sequencing technologies spearheaded by the long-read and portable platforms such as Oxford Nanopore (ONT) [17], *Plasmodium* WGS made possible by SWGA provides significant opportunities to study the genomic intricacies of malaria species. These insights include novel drug resistance mechanisms, genetic diversity, transmission dynamics and population structure [10]. WGS is being widely adopted in clinical settings and for disease surveillance for a variety of pathogens [17], and is expected to become increasingly valuable for patient management and clinical decisions.

Presently, the SWGA protocol deployed for studies of malaria parasites involves a lengthy step-down PCR process that includes an incubation step of 30 °C for 16 h [11], which follows the manufacturer's recommendations to compensate for non-specific DNase activity [18]. Although suited for research environments, where information is not generally under significant clinical time pressure, the prolonged incubation period will delay sequencing time and affect the feasibility of SWGA application in clinical care settings. Therefore, optimization of the SWGA strategy will be critical for timely WGS results returned in clinical or field-based settings. Moreover, MDA-facilitated genomic DNA amplification has been shown to plateau past 6 h [13]. This decrease suggests that, besides risking potential DNase degradation, the reduction of the additional time should not affect the amount of amplified DNA. Alternatively, a newer version of *phi29* DNA polymerase, the enzyme responsible for the SWGA process, has been developed with improved amplification bias and reaction speeds, enabling more efficient DNA amplification [19].

Thus, using nanopore sequencing, this study aimed to optimize the SWGA protocol to adapt it to a pipeline for rapid clinical response. Optimal incubation times were evaluated for SWGA by assessing the impact of shorter incubation periods on sequencing yields and genome coverage using the current SWGA protocol, and also investigated the performance of a newer version of *phi29* DNA polymerase. Here, by trialling different timepoints on clinical samples with varying levels of parasite DNA, the potential for a marked reduction in incubation times was investigated, which may lend feasibility for future clinical diagnostic use.

## Methods

### Sample processing

Thirteen *P. falciparum* DNA samples were obtained from the UK Health Security Agency-Malaria Reference Laboratory (UKHSA-MRL), and screened by microscopy and species identification previously confirmed by nested PCR and qPCR assays based on established methods [20, 21]. The samples were sourced from UK-treated patients who had returned from travelling to West (n = 8) and Central/East (n = 5) Africa and anonymised. Samples were extracted from whole blood samples using the QIAsymphony SP instrument with the DSP DNA kit (Qiagen) and following manufacturer’s guidelines. All samples were stored at −20 °C till use. Before their use, samples were subjected to quantification on a DS-11 FX + Spectrophotometer/Fluorometer (DeNovix) with the Qubit^™^ 1X dsDNA High Sensitivity Assay Kit and qPCR assay.

### SWGA with New England BioLab’s phi29 DNA polymerase

Using the same methods, albeit with various incubation times as outlined in previous work [14, 22], SWGA was applied to each DNA sample using established primer sets [22]. To minimize any potential contamination, SWGA reactions were prepared in a laminar airflow cabinet, Faster TWO30 (Faster Air). In brief, 120 ng of gDNA was diluted in Milli-Q^®^ water, filtered by Milli-Q^®^ Integral 10 system (Merck), to a maximum volume of 30 µL. To make a 50 µL total reaction, 5 µL of 10 × *phi29* DNA Polymerase Reaction Buffer (New England BioLabs; NEB), 0.35 µL of Purified 100 × BSA (NEB), 0.5 µL of 250 µM *P. falciparum*-SWGA primer mix, 5µL of 10 mM dNTP (Roche), 6.15 µL of Nuclease-Free Water (NFW; Takara Bio), and 30 units of *phi29* DNA Polymerase (NEB; equivalent to 3 µL) were added to the diluted gDNA. The reaction was conducted on a thermocycler with the modified step-down protocol: incubation at 35 °C for 5 min, 34 °C for 10 min, 33 °C for 15 min, 32 °C for 20 min, 31 °C for 30 min, and 30 °C for either 2 h or 8 h or 16 h, *phi29* DNA Polymerase heat inactivate at 65 °C for 15 min before holding at 10 °C (Fig. S1). The two additional timepoints were selected in consideration of a standard clinical workflow and the accumulative time taken for the amplification and library preparation, for which 2 h would have allowed for a same-day process while 8 h would have allowed for an overnight process. After amplification, the samples were purified by a 1:1 ratio bead-wash with KAPA beads (Roche), following the manufacturer’s protocol, and eluted in 31 µL of EB buffer (Qiagen) and were quantified post-SWGA and after purification as described above. The total reagent cost per sample for the SWGA reaction was £6.59.

### SWGA with EquiPhi29^™^ DNA polymerase

Three samples were amplified using EquiPhi29^™^ DNA polymerase. Samples were selected to reflect the varying *P. falciparum* concentration as measured by qPCR. The protocol, adapted from the manufacturer’s, is as follows: 120 ng of gDNA sample was combined with 0.5 µL of 10 × EquiPhi29^™^ DNA Polymerase Reaction Buffer (ThermoFisher Scientific), 0.5 µL of 250 µM *P. falciparum*-SWGA primer mix, and sufficient NFW (Takara Bio) to make up a total 5 µL reaction and together, was denatured by heating at 95 °C for 3 min and placed on ice for 5 min. 1.5 µL of 10 × EquiPhi29^™^ DNA Polymerase Reaction Buffer (ThermoFisher Scientific), 0.2 µL of 100 mM DTT (ThermoFisher Scientific), 2 µL of 10 mM dNTP (Roche), 10 units of EquiPhi29^™^ DNA Polymerase (ThermoFisher Scientific; equivalent to 1 µL), and 10.3 µL of NFW (Takara Bio) was added to the denatured solution. The solution was incubated at 45 °C for 3 h followed by 65 °C for 10 min to inactivate the polymerase and held at 4 °C. Amplified samples were likewise purified by bead-wash and quantified as outlined above with the final elution in 31 µL of EB buffer (Qiagen). The total reagent cost per sample for the SWGA reaction was £2.28.

### Library preparation and WGS

25.5 µL of amplified DNA first underwent T7 Endonuclease treatment to linearize and debranch the amplified material for the nanopore sequencing library preparation. Briefly, 25.5 µL of each DNA sample is combined with 3 µL of NEBuffer^™^ 2 (NEB) and 1.5 µL of T7 Endonuclease I (NEB). The solution is incubated at 37 °C for 15 min before undergoing a 0.6 × bead-wash purification with KAPA beads (Roche). DNA is eluted in 27 µL of NFW (Takara Bio) and incubated for 1 min at 50 °C and 5 min at room temperature. Purified DNA was similarly quantified as before, with 120 ng of DNA per sample taken forward for library preparation. DNA libraries were prepared using the Native Barcoding kit 24 V14 (SQK-NBD114.24, Oxford Nanopore Technologies (ONT)), according to the manufacturer’s protocol. Following ONT’s recommendations, 20 fmol of each library was sequenced for 22 h with R10.4.1 Flow cells on the MinION Mk1B platform (ONT). Sample sequences were demultiplexed by MinKNOW v23.07.12 (ONT) during the sequencing run. Technical repeats were performed for all samples that underwent SWGA with EquiPhi29^™^ DNA polymerase and for two samples that used the original *phi29* DNA polymerase, but only for the 8 h and 16 h timepoints (Figure S1, Table S3, Table S4). These replicates were run on new sequencing runs using the same amount of input DNA.

### Bioinformatic analysis for sequencing metrics, SNP variants, and drug resistance profiles

Raw sequence data (fasta files) were processed using a bioinformatic pipeline that includes *guppy* v6.5.7 (ONT) software for basecalling, *minimap2* (v2.26-r1175) for reference-assembly [23], *kraken2* (v2.1.3) for taxonomical identification [24], and *pycoQC* (v2.5.2) for data quality control [25] with the reference genome used being *P. falciparum* strain 3D7 (*Pf3D7*) (v2015-06-18) (PlasmoDB; https://plasmodb.org/plasmo/app). Using the *samtools* depth function on alignment bam files [26], sequence coverage was tabulated. Variant calling was performed by *FreeBayes* software (v1.3.2), with the following settings: minimum of 10 alternate allele counts, minimum coverage of tenfold depth, minimum base quality of 10, and disabling of clumping [27]. The diploid model can detect low frequency mutations, which is crucial for identifying mixed infections [28]. This would also accommodate for the possibility of multiclonal *P. falciparum* infections.

Pairwise SNP allele concordance between samples at different timepoints (8 h and 16 h) and allelic proportions (≥ 70%, ≥ 80%, or ≥ 90% alternative allele) was calculated across core genome positions [29]. Core genome positions were defined as the regions of the nuclear genome excluding the highly variable subtelomeric regions. A scoring system was developed to determine the alternate variants at various depths (10- to 30-fold), based on the proportion of alternate and reference alleles, while simulating potential *P. falciparum* clonal mixtures by varying the upper thresholds (ranging from 70 to 90%) required to classify the genotype call due to the potential clinical variability in samples. SNPs with allelic proportions below 50% were classified as reference genotypes, while those that exceeded the upper threshold were considered alternate genotypes. Allelic proportions between these thresholds were classified as mixed genotype calls. Concordance between the SNPs was determined by whether both positions shared the same genotype classification (alternate, reference, or mixed) and variant call. A 10% absolute difference threshold in allele proportions was permitted between mismatched classifications to account for sequencing error rate. This scoring system was developed to mitigate for genotype discrepancies observed from the *FreeBayes* output, which has been documented to produce false variant calls despite overwhelming variant counts to suggest otherwise [30]. However, it is important to note that minor clones may be excluded. Percentage concordance was calculated by the total number of concordant allelic variants divided by the total number of called positions. To control for variation in the number of sequenced reads between timepoints and samples of the two different protocols, reads from fastq files were randomly selected using *Rasusa* (v2.0.0), and normalized to the number of reads obtained by their corresponding 16 h timepoint [31]. Numbers selected were dependent on the comparison group. The reads were re-processed (e.g., mapped) as above. Cohen’s Kappa was used to assess the level of agreement between categorical genotype calls.

For drug resistance profiles, all raw fastq files were subsequently submitted to the online Malaria-Profiler platform [28] (https://bioinformatics.lshtm.ac.uk/malaria-profiler/) with options for ONT data, a soft cutoff depth of fivefold, a soft cutoff for minimum allele frequency at 50%, and all other parameters as default. During sample comparison, missing resistance mutations were flagged and these were inspected visually using the Integrative Genomics Viewer (v2.19.4) [32] to determine their status as present but under the depth threshold or lack of coverage over the position.

## Results

### Sample characteristics

Thirteen *P. falciparum*-positive DNA samples (Table 1) used in this study were from individuals who have reported travel between 2019 and 2020 to the following 10 countries: Nigeria (n = 2), Côte d'Ivoire (n = 2), Sudan (n = 2), Kenya (n = 1), Mali (n = 1), Congo (n = 1), Ghana (n = 1), Sierra Leone (n = 2), and Uganda (n = 1). *Plasmodium falciparum* mono-species infection was confirmed by qPCR and detected cycle threshold (Ct) values ranged from 13.01 to 36.43. Several samples (n = 7) exceeded a Ct threshold of 35, representing values with very low *Plasmodium* DNA. These latter samples remained included in this study due to prior confirmation of *P. falciparum* by the UKHSA-MRL.

**Table 1:** Characteristics of the 13 study samples

Sample ID	Country of origin	Year	Ct value
N1	Nigeria	2020	24.61
N2	Nigeria	2020	> 35
IC1	Cote d'Ivoire	2019	13.01
IC2	Cote d'Ivoire	2019	36.43
S1	Sudan	2020	30.98
S2	Sudan	2020	> 35
K1	Kenya	2020	27.73
M1	Mali	2019	32.91
C1	Congo	2020	23.90
G1	Ghana	2020	> 35
SL1	Sierra Leone	2020	> 35
SL2	Sierra Leone	2020	> 35
U1	Uganda	2020	> 35

### Incubation for 8 h produces similar SWGA enrichment and sequencing yields

**Fig. 1:**
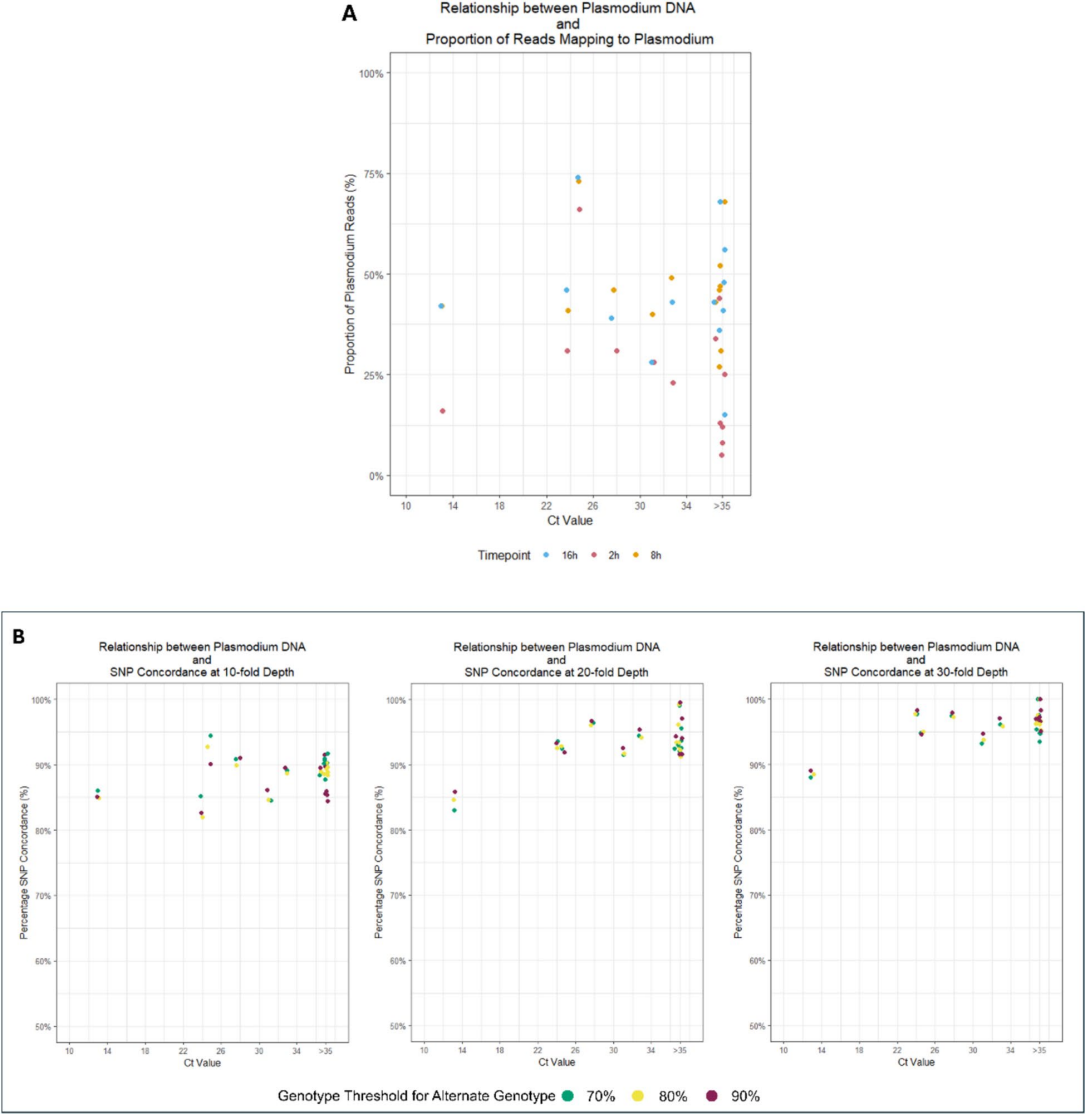
Correlation between starting *Plasmodium* DNA extracted from clinical whole blood samples and reads mapping to *Plasmodium* spp (**A**) and SNP concordance (**B**)

SWGA was performed with 3 different incubation times (2 h, 8 h, and 16 h) in its step-down PCR process, followed by a library preparation and sequencing using the nanopore platform for all samples. SWGA enrichment was observed to have similar effects for samples incubated for 8 h and 16 h, sharing matching SWGA enrichment results and sequencing yields. For SWGA enrichment, both timepoints had only differences of 0.1% and 2% in mean proportion of reads mapping to human and *Plasmodium* reference genomes, respectively. Individual sample analysis revealed a wide range in *Plasmodium* genus read proportions, from no difference up to a 12% increase. Notably, four 8-h timepoint samples showed a 10–12% higher proportion of *Plasmodium* genus reads compared to the 16-h timepoint (Fig. 1). However, unlike the 8 h timepoint, the 2 h timepoint showed poorer performance for enrichment with slightly more than half the proportion of *Plasmodium* genus reads obtained when compared to the 16 h timepoint. Based on DNA concentration, the 16 h timepoint was observed to still maintain the overall greater DNA enrichment yields, achieving 19.0 ng/µL and 6.4 ng/µL more in mean DNA concentration compared to the 2 h and 8 h timepoints, respectively (Table 2). However, in relation to the percentage of reads mapped to *P. falciparum* genome, both 8 h and 16 h timepoints had a similar proportion of *Plasmodium* reads (> 40% of total reads), while the 2 h timepoints had the lowest (~ 25.8%).

**Table 2:** Summary for sequencing yield of all samples (n = 13) across three time points

Incubation timepoint	2 h*	8 h*	16 h*
Mean DNA concentration after SWGA (ng/µL)	16.6 (7.0)	29.2 (14.7)	35.6 (18.9)
% of reads mapping to *Plasmodium*	25.8% (16.5%)	46.5% (12.7%)	44.5% (15.4%)
Total no. of reads mapping to *Plasmodium*	318,379 (227,060)	376,564 (189,639)	281,779 (182,428)
% of reads mapping to human	56.2% (18.2%)	29.5% (15.3%)	29.6% (19.5%)
% Core genome coverage at ≥ fivefold depth including apicoplast and mitochondria	21.23% (22.21%)	41.50% (23.57%)	38.89% (25.44%)
% Core genome coverage at ≥ fivefold depth excluding apicoplast and mitochondria	20.07% (20.68%)	42.02% (22.59%)	39.49% (24.66%)
Mean base quality	27.3 (1.7)	26.5 (1.3)	26.4 (1.0)

The DNA polymerase used was the original *phi29* DNA polymerase (NEB)

^*^Mean (SD); normalization was performed relative to the total number of sequenced reads

When examining core genome coverage at ≥ fivefold depth, samples from the 8-h timepoint generally performed like those from the 16-h timepoint, except for three samples in which the 8-h timepoint outperformed the others (Table S1) at a difference in mean coverage of 12.9%. As these observations were suspected to be driven by differences in total read counts (Table 2), the reads were normalized from all 8-h timepoint samples to match their 16-h counterparts and observed nearly identical coverage, with a mean difference of only 7.3% between timepoints. The subsequent exclusion of apicoplast and mitochondria genomes did not induce any drastic changes in the coverage (Table 2).

For the 2 h timepoint samples, normalization to the total number of reads sequenced at 16 h revealed a 19.1% lower mean percentage coverage compared to the 16 h timepoints. Furthermore, these samples showed poorer DNA amplification and *Plasmodium* enrichment, evidenced by the highest proportion of human reads and the lowest proportion of *Plasmodium* genus reads (Table 2). As a result, no further analyses were performed on the 2 h timepoint samples.

### SNP concordance between timepoints showed consistently high agreement

**Fig. 2:**
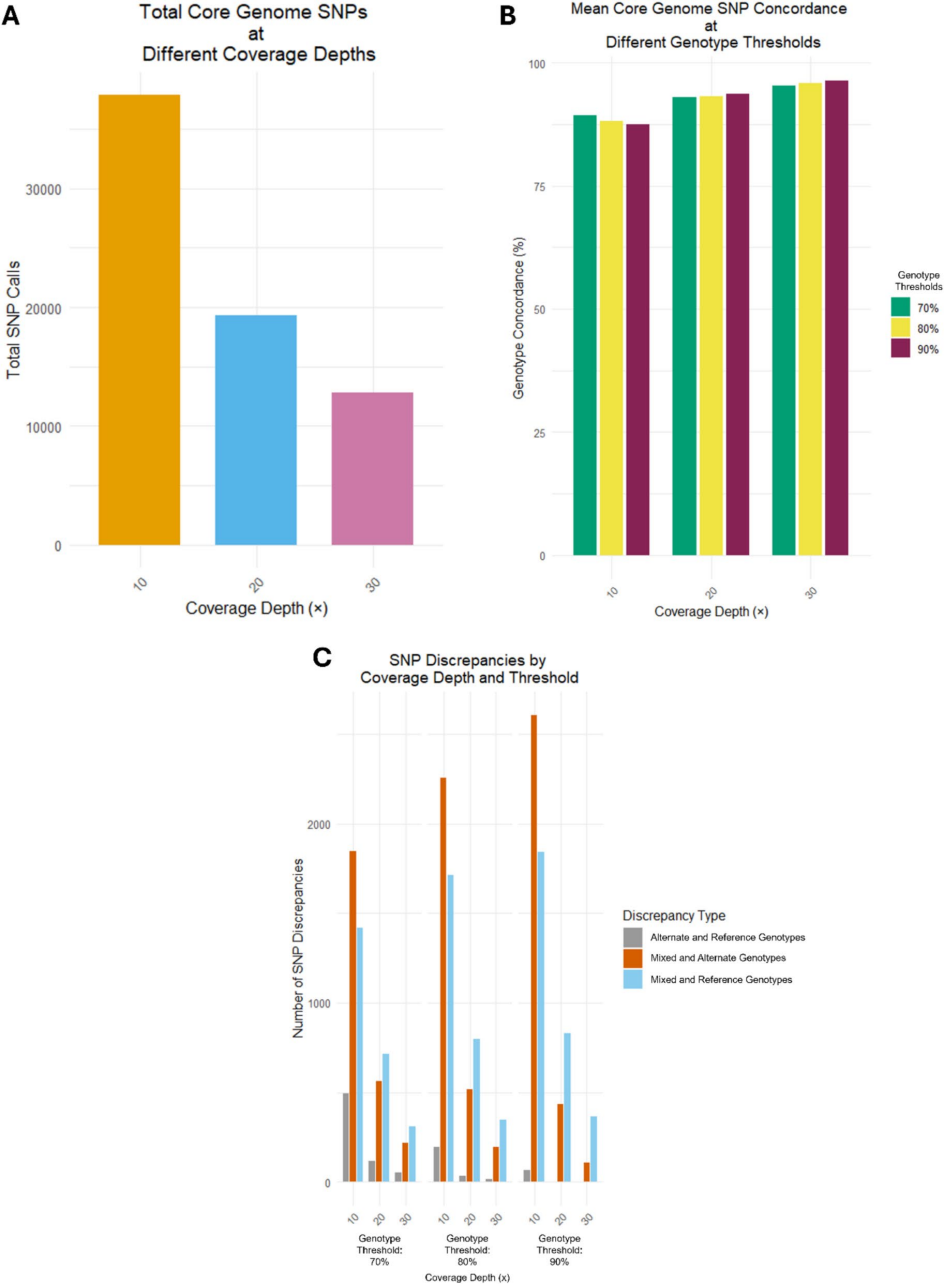
Visualization of SNP metrics for different SWGA incubation durations, including **A** total number of core genome SNPs called at each depth (tenfold, 20-fold, 30-fold), **B** the average SNP concordance at different genotype thresholds, and **C** the total number of SNP discrepancies arranged by discrepancy type

The mean percentage SNP concordance between timepoints ranged from 87.45 to 96.41% (Fig. 2, Table 3), with Cohen’s Kappa values ranging from 0.67 to 1.00. The highest concordance was observed at a 90% scoring threshold for clonal infections at 30-fold depth. As expected, SNP concordance increased with sequencing depth, exceeding 90% at 20-fold depth across all tested thresholds. Discordance was primarily observed between mixed genotypes and technical replicates, with alternate or reference genotypes being called differently; mixed-genotype discrepancies predominated at tenfold depth, while alternate/reference discrepancies became more common with increasing depth (Fig. 2, Table 3). Overall, these discordances were minor and were largely resolved at 30-fold depth (Table S2).

**Table 3:** Mean core genome SNP concordance at various variant thresholds and depths for all samples (n = 13)

Threshold for determining genotype concordance*		Coverage depth		
		tenfold	20-fold	30-fold
Genotype concordance	70% Alternate reads of total depth	89.26%	93.07%	95.41%
	80% Alternate reads of total depth	88.23%	93.13%	95.91%
	90% Alternate reads of total depth	87.45%	93.63%	96.41%
Total SNPs at given depth		37,888	19,344	12,846
SNP discrepancies at given threshold	Type of discrepancy			
70% alternate reads of total depth	Mixed and alternate calls	1,847	565	219
	Mixed and reference calls	1,417	715	312
	Alternate and reference calls	492	118	51
80% alternate reads of total depth	Mixed and alternate calls	2,257	515	193
	Mixed and reference calls	1,714	799	346
	Alternate and reference calls	195	32	14
90% alternate reads of total depth	Mixed and alternate calls	2,605	434	109
	Mixed and reference calls	1,842	828	363
	Alternate and reference calls	67	3	0

The DNA polymerase used was the original *phi29* DNA polymerase (NEB)

^*^Threshold refers to the proportion of alternate allele calls for an allele position. Allele proportions that fall between 50% to the upper threshold indicated in the table are considered mixed clones and those that fall below 50%, reference clones

#### SWGA protocol with EquiPhi29^™^ outperforms current protocol

To explore additional avenues to shorten protocol time, a different version of the *phi29* DNA polymerase that allows for a shorter incubation period was used. The manufacturer’s protocol was adapted to accommodate the primer set and applied the modified SWGA protocol to three samples (C1, IC1, M1) representative of the study set. Here, the results demonstrated that the new protocol surpassed, if not equal, those of the current approach (Table 4, Fig. S2).

**Table 4:** Individual sample comparison between SWGA results from the current and EquiPhi29^™^ SWGA protocol

Sample	IC1			C1			M1		
DNA polymerase used	*Phi29* (16 h)	*Phi29* (8 h)	EquiPhi29™	*Phi29* (16 h)	*Phi29* (8 h)	EquiPhi29™	*Phi29* (16 h)	*Phi29* (8 h)	EquiPhi29™
DNA concentration post-SWGA (ng/µL)	9.1	6.7	> 120*	13.6	14.8	> 120*	48.3	43.5	> 120*
Total number of reads	405,521	379,987	867,063	352,468	499,970	977,688	250,989	222,371	911,101
Mean read length	2,078.1	1,964.3	1,862.6	1,092.1	1,671.8	1,293.6	1,035.7	945.7	1,393.1
Read length N50	2,783.0	2,598.0	3,025.0	1,335.0	2,428.0	1,733.0	1,199.0	1,066.0	2,060.0
Proportion of reads mapped to *Plasmodium*	42%	42%	46%	46%	41%	50%	43%	49%	51%
Mean base quality	26.6	26.5	27.8	25.0	27.3	27.5	25.8	26.0	27.4
Percentage core genome coverage including apicoplast and mitochondria at ≥ fivefold depth†	76.67%	74.45%	79.29%	55.21%	72.90%	72.69%	25.47%	25.95%	42.18%
Percentage core genome coverage including apicoplast and mitochondria at ≥ tenfold depth†	50.30%	47.00%	57.83%	28.04%	42.20%	44.68%	11.08%	11.33%	24.43%

^*^Note that Qubit^™^ 1X dsDNA High Sensitivity Assay Kit has a maximum measuring limit of 120 ng

^†^All sample results including those on the second tested SWGA protocol were normalized by total read numbers to 16 h timepoints by random sampling using *rasusa*

**Fig. 3:**
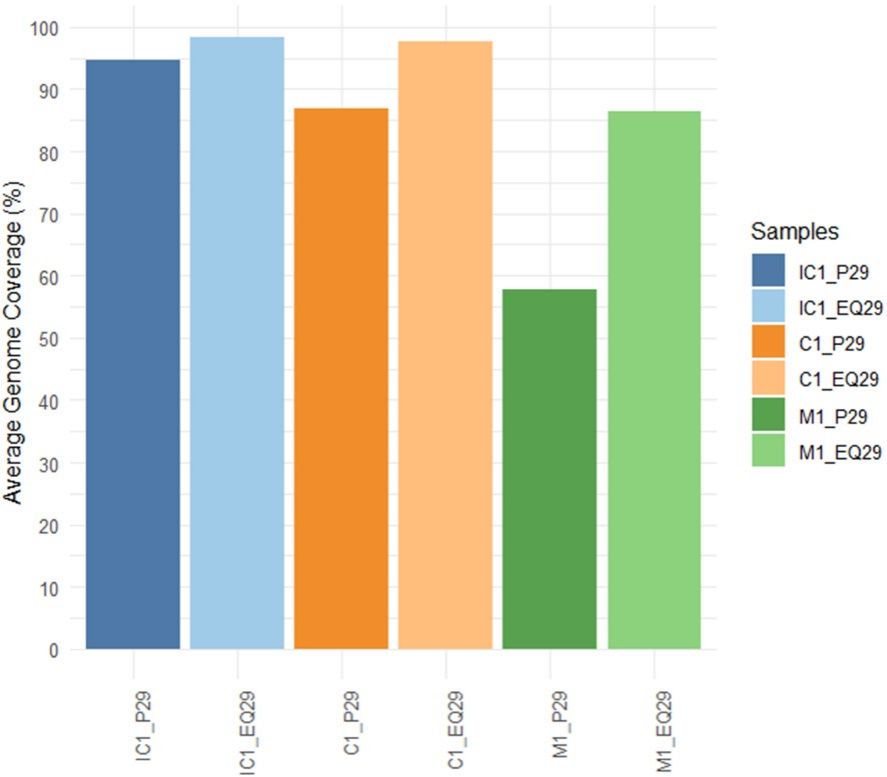
Average genome coverage achieved by each protocol for three different samples. 'P29' refers to the current *phi29* DNA polymerase used in the existing protocol, while 'EQ29' refers to EquiPhi29^™^, used in the second tested SWGA protocol

Firstly, a 3 h incubation period outlined in the manufacturer’s protocol was utilized, which is shorter than the total incubation period of over 16 h used in the current SWGA protocol. This enabled the processing and sequencing of the DNA samples within the same day. Secondly, the SWGA enrichment using the new protocol yielded a greater total DNA output (Table 4). With the exception of sample M1, this was associated with a slightly higher proportion of *P. falciparum*-mapped reads and a lower proportion of human-mapped reads. Third, the increased mapping resulted in higher *P. falciparum* genome coverage and mean depth, with the largest gains being a 28.8% increase in coverage and a 2.7- to 5.3-fold increase in depth (Fig. 3, Table 4). Lastly, mean base quality was either slightly improved or maintained following the SWGA protocol. These results highlighted that the new protocol did not compromise the base calling accuracy. As the total number of reads exceeded that of the current protocol, the reads were normalized again to both those obtained by the SWGA 8 h and 16 h timepoint samples. Likewise, the percentage coverage at both ≥ fivefold depth and ≥ tenfold depth was greater for all samples regardless of timepoints (Table 4).

When analysing SNP concordance across varying thresholds, normalized results for both 8 h and 16 h timepoints were used. Overall concordance ranged from 70.33 to 89.42% for the 16 h timepoint comparisons and from 69.75 to 91.53% for the 8 h comparisons across all tested depths (Table 5). While discrepancies most commonly involved mixed and reference genotypes, there was an observed increase in discordance between mixed and alternate genotypes, which decreased with higher coverage depths. Consistent with previous findings, sample M1 contributed the majority of these discrepancies, accounting for 72.7% (750/1031) to 93.1% (522/561) of this type of discordance at ≥ tenfold depth (Table S5).

**Table 5:** Average SNP concordance at allelic proportion thresholds of 70%, 80%, and 90%, and coverage depths of 10x, 20x, and 30x, for three samples (IC1, C1, and M1) processed using the SWGA protocol utilizing EquiPhi29^™^ (ThermoFisher Scientific), compared to the same samples amplified using the original protocol at the 8 h and 16 h timepoints

Threshold for determining genotype concordance	Coverage depth					
	tenfold		20-fold		30-fold	
	8 h timepoint	16 h timepoint	8 h timepoint	16 h timepoint	8 h timepoint	16 h timepoint
70% Alternate reads of total depth	73.66%	74.71%	83.83%	82.52%	91.53%	89.42%
80% Alternate reads of total depth	71.77%	72.96%	82.89%	81.21%	90.46%	88.40%
90% Alternate reads of total depth	69.75%	70.33%	82.30%	79.77%	89.99%	87.61%
Total SNPs	10,464	11,405	3,389	3,978	1,712	2,001

The genotype concordances were measured by comparing the genotype calls obtained from the EquiPhi29^™^ SWGA protocol to the calls obtained from the current SWGA protocol using the original *phi29* (NEB) at incubation durations of 8 h and 16 h

For the technical replicates of the new protocol (IC1, C1, and M1), normalization of total read numbers resulted in a 5.10% difference in core genome coverage at ≥ fivefold depth (Table S6). SNP concordance across replicates ranged from 60.86% to 98.39% between ≥ tenfold and ≥ 30-fold depths when compared to their corresponding 8 h and 16 h timepoint samples. Notably, sample C1 exhibited 93.02% concordance with its 8-h timepoint sample at ≥ 20-fold depth (Table S7). Overall, replicate comparisons showed concordance up to 98.64%, with all samples exceeding 90% concordance at ≥ 20-fold depth, and sample M1 achieving nearly 95% concordance even at ≥ tenfold depth.

### High-level of drug resistance profiles consistency for all samples of both protocols

To ensure consistency in detecting potential drug resistances, raw sequencing files were analysed using *Malaria-Profiler* [28] to generate a drug resistance profile for each sample. Matching drug resistance profiles were obtained for 29 of the 32 samples across various conditions (Table 6). Ten samples were considered perfect matches, exhibiting identical sets of drug resistance mutations across different timepoints. Manual inspection of drug resistance loci in corresponding samples revealed additional mutations present below the soft cutoff threshold, which were therefore omitted from the automated profile reports. For the remaining three samples with discordant drug resistance profiles, differences were attributed not to the absence of mutations but to lack of sequencing coverage in the relevant regions.

**Table 6:**
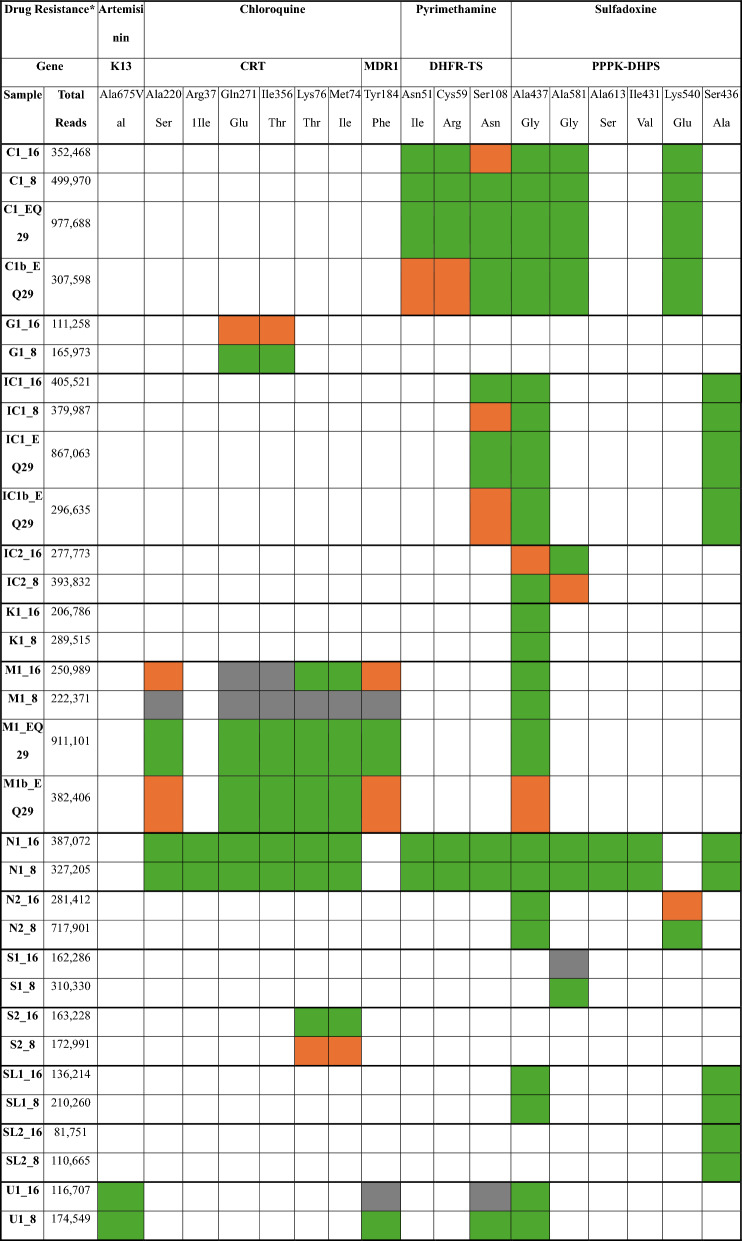
Drug resistance profiles of individual samples obtained from Malaria-Profiler. These are accompanied by the total number of reads, the genes affected, and the related protein mutations

^*^Colours for each cell represent the detection of the corresponding mutation in the samples: white means absent, green means present with 5 × coverage depth (n = 98), orange means present but < 5 × coverage depth (n = 17), and grey means no coverage over the area (n = 10)

## Discussion

SWGA is an important strategy for *Plasmodium* DNA enrichment, circumventing the biological challenges of performing WGS for malaria parasites. WGS is playing an increasingly important role in infection control decision-making in both clinical and surveillance settings. To extend the applicability of SWGA beyond research and into clinical use, the study aimed to optimize the protocol to deliver results as rapidly as possible, to better fit within clinically relevant timeframes. The work suggests that incubation times for processing of samples can be reduced from 16 to 8 h, while achieving excellent overall results, including optimum genome coverage and minimal interference from human DNA in the starting material. In addition, a new protocol with a shorter 3-h amplification was evaluated, which provided improved amplification efficiency, higher sequencing yields and data quality, and increased coverage—with at least 63.4% of the nuclear genome covered at ≥ 10 reads—at just over one-third of the reagent cost. This improvement in affordability and turnaround time will support clinical and surveillance genomics in malaria-endemic countries, enhancing routine investigations, outbreak responses, and the monitoring of resurgences driven by imported cases.

As with other published protocols [33, 34], ONT sequencing was utilized due to its operational feasibility, portability and accessibility [9, 10], with long read generation improving sequence assemblies [35]. However, this platform can introduce a higher sequencing error rate, especially around homopolymeric tracts [33], which are common in the *P. falciparum* genome [36–38]. This sequencing error rate offers a potential explanation for the ~ 10% average SNP discrepancy observed across all the compared samples. With previously documented instances of incorrect genotype compositions corresponding to low coverage [39], this could further explain the level of mixed and alternate or reference genotype discrepancies, as coverage achieved either from the 8 h and 16 h timepoints might not be sufficient for accurate variant calling. Moreover, SNP discrepancies could be additionally attributed to an SWGA error and should be further investigated with reference samples to distinguish between sequencing or amplification error.

Low coverage can induce SNP discordance with variants tending to the reference allele [33]. A decline in mixed and alternate allelic genotype discrepancies was observed with increasing coverage depths, indicating that some of such discordance might be avoided if greater coverage depths were established (> 30-fold). Notably, the discrepancy was higher for sample M1, which had the lowest detectable parasite DNA (Ct = 32.91, IC1 with Ct = 13.01, and C1 with Ct = 23.9). Similarly, other studies utilising SWGA have reported correlations between parasitaemia levels and SWGA efficiency, as well as inconsistencies between sample replicates, even when using the more precise Illumina sequencing platform [10, 34].

Genotypic discrepancies observed across the same SWGA samples amplified using different protocols or timepoints could also be partly attributed to the *FreeBayes* variant caller, which determines variants based on quality estimations derived from factors such as base quality [27]. Whilst the *FreeBayes* diploid setting had sufficient coverage to process positions of mismatch, there appears to be insufficient likelihood to call it an alternative allele due to the Bayesian a priori probabilities [40]. This situation has been observed when employing the diploid model, including by other variant callers such as *Clair3* [34]. Therefore, although this may affect the calling of minority genotypes, the scoring system was instituted with a stringent 50% allele proportion threshold, to negate any potential error-rate effects. Furthermore, to control for variability in the sequencing runs and different total read numbers, random subsampling was performed using the *rasusa* bioinformatics program. Random read sampling might skew certain results, although there were no observable changes in the proportion of reads mapping to *Plasmodium* and human genomes.

Importantly, the drug resistance profiles were consistent for almost all samples analysed by both protocols, indicating that WGS-based drug susceptibility profiling performs reliably under the SWGA methods implemented. Samples with more than 400,000 sequenced reads had more reliable drug resistance profiles generated, suggesting that this may be a suitable minimum threshold for the analysis of clinical isolates. Most of the samples had genotypic markers of drug resistance to pyrimethamine and sulfadoxine, which are widely reported across the African continent, due to their use as replacements for chloroquine before their own replacement by ACT [29]. Notably, the protocol detected an artemisinin resistance mutation *pfk13* A675V in a sample of Ugandan origin (U1), a mutation first reported in Northern Uganda in 2016 [41]. Its prevalence has risen sharply, from 2% in 2016–2017 to 41% in 2018–2019 [42, 43]. In the context of emerging artemisinin resistance across Africa and the high malaria burden in this region, the findings underscore the critical need for ongoing genomic surveillance and accessible screening tools to monitor drug resistance, particularly within endemic countries.

SWGA has inherent limitations due to its underlying process, which complicates the detection of copy number variations that are critical for profiling drug resistance, such as piperaquine and mefloquine resistance [44]. In addition, the primers are designed to select against mitochondrial genomes, potentially preventing detection of atovaquone resistance [22, 44]. Interestingly, considerable amplification of the apicoplast genome was observed when using EquiPhi29^™^, though analysis of apicoplast and mitochondrial genomes was not conducted given the primer design; this warrants further investigation once the underlying mechanism is better understood. The method used to assess SNP concordance may also underrepresent minor clonal populations, which can be clinically significant [45]. The limited number of samples and replicates constrains the generalizability of these conclusions, and future validation with larger sample sets will be important. Testing these protocols using dried blood spots will also be valuable. Nevertheless, this study provides promising evidence and trends supporting both the optimization of the current protocol and the new 3-h protocol.

The advancements in the SWGA protocol presented here will support the routine use of this method and WGS in clinical settings, particularly as novel drug resistance emerges and undermines the effectiveness of many treatment options. More broadly, the rising global antimalarial drug resistance underscores the need for molecular diagnostics and WGS to be integrated into national control programme policies [46–48]. While sequencing technologies may be perceived as costly, reductions in WGS costs, along with the development of amplicon-based sequencing and the improved SWGA protocol presented here, will enhance accessibility in endemic regions. Benefits will be most impactful when integrated with established surveillance programmes that incorporate data from therapeutic efficacy studies [44]. With advancing sequencing technologies, SWGA is poised to enable the transition of WGS from primarily a research and surveillance tool for malaria to one with direct clinical applications. Incubation at three different timepoints was evaluated and its impact on nanopore sequencing yield, genome coverage, and SNP concordance was also assessed. Furthermore, the modified SWGA protocol, which incorporates a fast-acting *phi29* DNA polymerase, outperforms current methods by reducing costs and turnaround time, thereby enhancing its potential for use in clinical settings where affordability and rapid results are essential.

## Supplementary Information

Supplementary material 1.

## Data Availability

The raw sequence data generated and analysed during the current study are available in the European Nucleotide Archive (Accession: PRJEB90161).

## Abbreviations

ACT: Artemisinin-based combination therapy
MDA: Multiple displacement amplification
NEB: New England BioLabs
NFW: Nuclease-free water
ONT: Oxford Nanopore
SP: Sulfadoxine-pyrimethamine
SWGA: Selective whole genome amplification
UKHSA-MRL: The UK Health Security Agency-Malaria Reference Laboratory
WGS: Whole genome sequencing
WHO: World Health Organization
