# Supplementary material for: Enhancing whole genome DNA amplification of Plasmodium falciparum for advanced molecular surveillance in malaria control
Source: Malar J. 2025 Dec 19;24:445. doi: 10.1186/s12936-025-05643-9 (PMC12717693; doi:10.1186/s12936-025-05643-9)

**Supplementary Information**

**Table S1.** Sequencing yield and metrics by individual samples processed with the current protocol. *Note that Qubit™ 1X dsDNA High Sensitivity Assay Kit has a maximum measuring limit of 120ng; for these samples 2µL of starting DNA was added.

| **Sample** | **Timepoint (h)** | **DNA Concentration (ng/µL)** | | **Total Number of Reads** | **Mean Read Length** | **Read Length N50** | **Proportion of Reads Mapped to *Plasmodium* (%)** | **Proportion of Reads Mapped to Human (%)** | **Percentage Core Genome Coverage including Apicoplast and Mitochondria at** ≥**5x Depth (%)** | | **Mean Base Quality** | **Mean Map Quality** |
| --- | --- | --- | --- | --- | --- | --- | --- | --- | --- | --- | --- | --- |
|  |  | **Before SWGA** | **After SWGA** |  |  |  |  |  | **Total Read Normalization to 16h Timepoint** | |  |  |
|  |  |  |  |  |  |  |  |  | **Before** | **After** |  |  |
| N1 | 2 | >120* | 2.6 | 885,737 | 1,077.0 | 1,161.0 | 66 | 24 | 92.46 | 74.86 | 30.6 | 57.0 |
|  | 8 |  | 7.5 | 327,205 | 1,682.7 | 2,084.0 | 73 | 10 | 92.27 | 92.27 | 26.6 | 57.0 |
|  | 16 |  | 9.1 | 387,072 | 1,711.2 | 2,220.0 | 74 | 7 | 94.08 | - | 26.6 | 56.9 |
| N2 | 2 | 83.2 | 21.2 | 983,632 | 1,402.7 | 1,922.0 | 8 | 78 | 19.68 | 3.11 | 24.1 | 55.8 |
|  | 8 |  | 13 | 717,901 | 1,558.4 | 2,172.0 | 27 | 40 | 51.57 | 26.16 | 28.3 | 53.1 |
|  | 16 |  | 14.2 | 281,412 | 1,111.6 | 1,391.0 | 15 | 67 | 10.27 | - | 26.8 | 56.2 |
| IC1 | 2 | >120* | 1.9 | 542,777 | 1,190.4 | 1,344.0 | 16 | 66 | 31.86 | 22.77 | 26.9 | 56.2 |
|  | 8 |  | 6.7 | 379,987 | 1,964.3 | 2,598.0 | 42 | 43 | 74.45 | 74.45 | 26.5 | 57.0 |
|  | 16 |  | 9.1 | 405,521 | 2,078.1 | 2,783.0 | 42 | 42 | 76.67 | - | 26.6 | 56.6 |
| IC2 | 2 | 64.5 | 16.2 | 289,736 | 1,063.1 | 1,271.0 | 34 | 34 | 25.04 | 24.02 | 25.0 | 51.2 |
|  | 8 |  | 36.8 | 393,832 | 1,100.8 | 1,304.0 | 43 | 19 | 41.53 | 31.58 | 23.3 | 50.3 |
|  | 16 |  | 55.8 | 277,773 | 1,043.8 | 1,208.0 | 43 | 10 | 27.92 | - | 25.5 | 53.2 |
| S1 | 2 | 45.6 | 19.5 | 314,533 | 960.0 | 1,108.0 | 28 | 42 | 21.40 | 9.89 | 26.9 | 54.8 |
|  | 8 |  | 38.8 | 310,330 | 1,046.6 | 1,254.0 | 40 | 26 | 33.35 | 17.67 | 26.4 | 54.8 |
|  | 16 |  | 43.9 | 162,286 | 960.6 | 1,081.0 | 28 | 17 | 4.75 | - | 27.0 | 49.0 |
| S2 | 2 | 58.7 | 19.6 | 838,266 | 1,115.8 | 1,394.0 | 25 | 61 | 89.94 | 31.68 | 26.3 | 55.6 |
|  | 8 |  | 15 | 172,991 | 967.2 | 1,176.0 | 52 | 25 | 28.80 | 27.03 | 25.0 | 54.1 |
|  | 16 |  | 14.2 | 163,228 | 1,017.5 | 1,248.0 | 56 | 18 | 36.11 | - | 27.5 | 55.6 |
| K1 | 2 | 54.7 | 17.1 | 314,805 | 1,087.3 | 1,319.0 | 31 | 43 | 29.42 | 18.55 | 26.3 | 55.6 |
|  | 8 |  | 36.3 | 289,515 | 952.4 | 1,084.0 | 46 | 9 | 31.87 | 23.41 | 26.6 | 52.9 |
|  | 16 |  | 51.6 | 206,786 | 942.7 | 1,059.0 | 39 | 10 | 15.37 | - | 24.5 | 51.8 |
| M1 | 2 | 73.7 | 16.8 | 411,619 | 1,057.1 | 1,272.0 | 23 | 52 | 23.94 | 13.93 | 27.4 | 55.5 |
|  | 8 |  | 43.5 | 222,371 | 945.7 | 1,066.0 | 49 | 12 | 25.95 | 25.95 | 26.0 | 55.1 |
|  | 16 |  | 48.3 | 250,989 | 1,035.7 | 1,199.0 | 43 | 13 | 25.47 | - | 25.8 | 53.1 |
| C1 | 2 | >120* | 24 | 878,101 | 1,759.4 | 2,618.0 | 31 | 57 | 89.67 | 56.15 | 26.9 | 56.7 |
|  | 8 |  | 14.8 | 499,970 | 1,671.8 | 2,428.0 | 41 | 43 | 83.91 | 72.90 | 27.3 | 56.7 |
|  | 16 |  | 13.6 | 352,468 | 1,092.1 | 1,335.0 | 46 | 41 | 55.21 | - | 25.0 | 56.9 |
| G1 | 2 | >120* | 27.4 | 394,005 | 1,923.8 | 2,766.0 | 12 | 76 | 29.74 | 2.35 | 28.2 | 55.8 |
|  | 8 |  | 41.6 | 165,973 | 2,133.9 | 3,021.0 | 47 | 39 | 54.16 | 37.62 | 27.6 | 56.0 |
|  | 16 |  | 54.7 | 111,258 | 2,338.1 | 3,985.0 | 48 | 41 | 40.26 | - | 26.4 | 56.8 |
| SL1 | 2 | >120* | 17.8 | 572,881 | 1,450.0 | 1,911.0 | 5 | 81 | 10.26 | 0.57 | 28.7 | 55.3 |
|  | 8 |  | 34.5 | 210,260 | 2,423.1 | 3,712.0 | 31 | 57 | 42.59 | 26.35 | 26.9 | 56.7 |
|  | 16 |  | 54 | 136,214 | 2,675.1 | 4,563.0 | 41 | 47 | 41.13 | - | 26.9 | 57.1 |
| SL2 | 2 | 52.4 | 18.2 | 555,326 | 1,405.0 | 1,852.0 | 44 | 44 | 80.66 | 16.24 | 29.2 | 56.8 |
|  | 8 |  | 50.1 | 110,665 | 2,201.6 | 3,117.0 | 68 | 19 | 61.52 | 48.85 | 26.9 | 56.9 |
|  | 16 |  | 47.3 | 81,751 | 2,470.3 | 4,075.0 | 68 | 20 | 48.46 | - | 26.6 | 56.7 |
| U1 | 2 | 62.3 | 14 | 487,384 | 1,339.0 | 1,685.0 | 13 | 73 | 21.60 | 1.80 | 28.4 | 56.4 |
|  | 8 |  | 40.8 | 174,549 | 2,164.7 | 3,229.0 | 46 | 41 | 50.73 | 35.30 | 27.5 | 57.0 |
|  | 16 |  | 46.7 | 116,707 | 2,777.6 | 4,646.0 | 36 | 52 | 29.80 | - | 28.3 | 56.5 |

**Table S2.** SNP concordance by samples, grouped by allelic proportion thresholds and including individual discrepancies breakdown. MA = Mixed and Alternate calls, MR = Mixed and Reference calls, and AR = Alternate and Reference calls.

| **Sample** | **Coverage Depth**  **(-fold)** | **70% Alternative Reads of Total Depth** | | | | | | |
| --- | --- | --- | --- | --- | --- | --- | --- | --- |
|  |  | **Total SNPs Called** | **Concordant SNPs** | **Percentage Concordance (%)** | **Cohen Kappa** | **Type of Discrepancy** | | |
|  |  |  |  |  |  | **MA** | **MR** | **AR** |
| N1 | 10 | 9,793 | 9,249 | 94.45 | 0.77 | 341 | 170 | 26 |
|  | 20 | 4,517 | 4,177 | 92.47 | 0.81 | 166 | 150 | 17 |
|  | 30 | 2,092 | 1,984 | 94.84 | 0.90 | 37 | 61 | 4 |
| N2 | 10 | 693 | 626 | 90.33 | 0.78 | 26 | 30 | 11 |
|  | 20 | 428 | 424 | 99.07 | 0.95 | 2 | 1 | 1 |
|  | 30 | 385 | 385 | 100 | 1 | 0 | 0 | 0 |
| IC1 | 10 | 2,873 | 2,473 | 86.08 | 0.74 | 197 | 174 | 29 |
|  | 20 | 1,498 | 1,244 | 83.04 | 0.74 | 85 | 154 | 15 |
|  | 30 | 760 | 669 | 88.03 | 0.79 | 20 | 68 | 3 |
| IC2 | 10 | 4,239 | 3,749 | 88.44 | 0.80 | 238 | 191 | 61 |
|  | 20 | 3,024 | 2,795 | 92.43 | 0.83 | 98 | 113 | 18 |
|  | 30 | 2,480 | 2,367 | 95.44 | 0.87 | 50 | 57 | 6 |
| S1 | 10 | 2,077 | 1,756 | 84.55 | 0.67 | 118 | 126 | 77 |
|  | 20 | 1,624 | 1,486 | 91.50 | 0.75 | 39 | 77 | 22 |
|  | 30 | 1,316 | 1,227 | 93.24 | 0.77 | 32 | 41 | 16 |
| S2 | 10 | 2,440 | 2,141 | 87.75 | 0.75 | 118 | 134 | 47 |
|  | 20 | 1,144 | 1,094 | 95.63 | 0.85 | 18 | 23 | 9 |
|  | 30 | 782 | 763 | 97.57 | 0.89 | 7 | 10 | 2 |
| K1 | 10 | 2,914 | 2,649 | 90.91 | 0.83 | 146 | 94 | 25 |
|  | 20 | 2,151 | 2,074 | 96.42 | 0.90 | 34 | 37 | 6 |
|  | 30 | 1,891 | 1,844 | 97.51 | 0.92 | 21 | 23 | 3 |
| M1 | 10 | 3,152 | 2,812 | 89.21 | 0.81 | 149 | 135 | 56 |
|  | 20 | 2,194 | 2,072 | 94.44 | 0.86 | 47 | 62 | 13 |
|  | 30 | 1,831 | 1,762 | 96.18 | 0.88 | 27 | 31 | 11 |
| C1 | 10 | 2,591 | 2,208 | 85.21 | 0.75 | 170 | 137 | 76 |
|  | 20 | 920 | 861 | 93.59 | 0.87 | 19 | 34 | 6 |
|  | 30 | 534 | 522 | 97.75 | 0.94 | 5 | 7 | 0 |
| G1 | 10 | 1,688 | 1,535 | 90.94 | 0.76 | 78 | 52 | 23 |
|  | 20 | 429 | 402 | 93.71 | 0.87 | 11 | 14 | 2 |
|  | 30 | 149 | 144 | 96.64 | 0.94 | 3 | 2 | 0 |
| SL1 | 10 | 1,683 | 1,543 | 91.68 | 0.79 | 86 | 36 | 18 |
|  | 20 | 474 | 441 | 93.04 | 0.87 | 19 | 11 | 3 |
|  | 30 | 208 | 197 | 94.71 | 0.90 | 5 | 3 | 3 |
| SL2 | 10 | 2,298 | 2,072 | 90.17 | 0.70 | 112 | 91 | 23 |
|  | 20 | 525 | 486 | 92.57 | 0.85 | 16 | 21 | 2 |
|  | 30 | 234 | 222 | 94.87 | 0.90 | 7 | 5 | 0 |
| U1 | 10 | 1,447 | 1,312 | 90.67 | 0.78 | 68 | 47 | 20 |
|  | 20 | 416 | 383 | 92.07 | 0.86 | 11 | 18 | 4 |
|  | 30 | 184 | 172 | 93.48 | 0.88 | 5 | 4 | 3 |
|  | | | | | | | | |
| **Sample** | **Coverage Depth**  **(-fold)** | **80% Alternative Reads of Total Depth** | | | | | | |
|  |  | **Total SNPs Called** | **Concordant SNPs** | **Percentage Concordance (%)** | **Cohen Kappa** | **Type of Discrepancy** | | |
|  |  |  |  |  |  | **MA** | **MR** | **AR** |
| N1 | 10 | 9,793 | 9,087 | 92.79 | 0.77 | 503 | 192 | 4 |
|  | 20 | 4,517 | 4,194 | 92.85 | 0.85 | 149 | 165 | 2 |
|  | 30 | 2,092 | 1,988 | 95.03 | 0.91 | 33 | 65 | 0 |
| N2 | 10 | 693 | 626 | 90.33 | 0.78 | 26 | 35 | 6 |
|  | 20 | 428 | 425 | 99.30 | 0.97 | 1 | 2 | 0 |
|  | 30 | 385 | 385 | 100 | 1 | 0 | 0 | 0 |
| IC1 | 10 | 2,873 | 2,439 | 84.89 | 0.75 | 231 | 191 | 12 |
|  | 20 | 1,498 | 1,268 | 84.65 | 0.77 | 61 | 165 | 4 |
|  | 30 | 760 | 673 | 88.55 | 0.80 | 16 | 71 | 0 |
| IC2 | 10 | 4,239 | 3,770 | 88.94 | 0.82 | 217 | 239 | 13 |
|  | 20 | 3,024 | 2,824 | 93.39 | 0.86 | 69 | 128 | 3 |
|  | 30 | 2,480 | 2,386 | 96.21 | 0.89 | 31 | 63 | 0 |
| S1 | 10 | 2,077 | 1,758 | 84.64 | 0.67 | 116 | 166 | 37 |
|  | 20 | 1,624 | 1,489 | 91.69 | 0.76 | 36 | 92 | 7 |
|  | 30 | 1,316 | 1,234 | 93.77 | 0.79 | 25 | 52 | 5 |
| S2 | 10 | 2,440 | 2,162 | 88.61 | 0.77 | 97 | 161 | 20 |
|  | 20 | 1,144 | 1,100 | 96.15 | 0.87 | 12 | 29 | 3 |
|  | 30 | 782 | 763 | 97.57 | 0.89 | 7 | 10 | 2 |
| K1 | 10 | 2,914 | 2,619 | 89.88 | 0.81 | 176 | 111 | 8 |
|  | 20 | 2,151 | 2,067 | 96.09 | 0.89 | 41 | 42 | 1 |
|  | 30 | 1,891 | 1,839 | 97.25 | 0.91 | 26 | 26 | 0 |
| M1 | 10 | 3,152 | 2,795 | 88.67 | 0.80 | 166 | 164 | 27 |
|  | 20 | 2,194 | 2,066 | 94.17 | 0.86 | 53 | 67 | 8 |
|  | 30 | 1,831 | 1,756 | 95.90 | 0.87 | 33 | 35 | 7 |
| C1 | 10 | 2,591 | 2125 | 82.01 | 0.72 | 253 | 181 | 32 |
|  | 20 | 920 | 852 | 92.61 | 0.85 | 28 | 37 | 3 |
|  | 30 | 534 | 522 | 97.75 | 0.94 | 5 | 7 | 0 |
| G1 | 10 | 1,688 | 1,509 | 89.40 | 0.76 | 104 | 60 | 15 |
|  | 20 | 429 | 398 | 92.77 | 0.88 | 15 | 13 | 1 |
|  | 30 | 149 | 144 | 96.64 | 0.94 | 3 | 2 | 0 |
| SL1 | 10 | 1,683 | 1,508 | 89.60 | 0.77 | 121 | 46 | 8 |
|  | 20 | 474 | 443 | 93.46 | 0.89 | 17 | 14 | 0 |
|  | 30 | 208 | 201 | 96.63 | 0.94 | 1 | 6 | 0 |
| SL2 | 10 | 2,298 | 2,031 | 88.38 | 0.70 | 153 | 106 | 8 |
|  | 20 | 525 | 479 | 91.24 | 0.84 | 23 | 23 | 0 |
|  | 30 | 234 | 223 | 95.30 | 0.92 | 6 | 5 | 0 |
| U1 | 10 | 1,447 | 1,286 | 88.87 | 0.77 | 94 | 62 | 5 |
|  | 20 | 416 | 384 | 92.31 | 0.87 | 10 | 22 | 0 |
|  | 30 | 184 | 177 | 96.20 | 0.93 | 7 | 4 | 0 |
|  | | | | | | | | |
| **Sample** | **Coverage Depth**  **(-fold)** | **90% Alternative Reads of Total Depth** | | | | | | |
|  |  | **Total SNPs Called** | **Concordant SNPs** | **Percentage Concordance (%)** | **Cohen Kappa** | **Type of Discrepancy** | | |
|  |  |  |  |  |  | **MA** | **MR** | **AR** |
| N1 | 10 | 9,793 | 8,828 | 90.15 | 0.77 | 762 | 196 | 0 |
|  | 20 | 4,517 | 4,151 | 91.90 | 0.85 | 192 | 167 | 0 |
|  | 30 | 2,092 | 1,981 | 94.69 | 0.91 | 40 | 65 | 0 |
| N2 | 10 | 693 | 634 | 91.49 | 0.80 | 18 | 39 | 2 |
|  | 20 | 428 | 426 | 99.53 | 0.98 | 2 | 0 | 0 |
|  | 30 | 385 | 385 | 100 | 1 | 0 | 0 | 0 |
| IC1 | 10 | 2,873 | 2,445 | 85.10 | 0.77 | 225 | 200 | 3 |
|  | 20 | 1,498 | 1,286 | 85.85 | 0.79 | 43 | 167 | 2 |
|  | 30 | 760 | 677 | 89.08 | 0.81 | 12 | 71 | 0 |
| IC2 | 10 | 4,239 | 3,796 | 89.55 | 0.83 | 191 | 249 | 3 |
|  | 20 | 3,024 | 2,855 | 94.41 | 0.88 | 38 | 131 | 0 |
|  | 30 | 2,480 | 2,406 | 97.02 | 0.91 | 11 | 63 | 0 |
| S1 | 10 | 2,077 | 1,789 | 86.13 | 0.70 | 85 | 184 | 19 |
|  | 20 | 1,624 | 1,504 | 92.61 | 0.78 | 21 | 99 | 0 |
|  | 30 | 1,316 | 1,247 | 94.76 | 0.82 | 12 | 57 | 0 |
| S2 | 10 | 2,440 | 2,189 | 89.71 | 0.80 | 70 | 177 | 4 |
|  | 20 | 1,144 | 1,111 | 97.12 | 0.90 | 1 | 32 | 0 |
|  | 30 | 782 | 769 | 98.34 | 0.93 | 1 | 12 | 0 |
| K1 | 10 | 2,914 | 2,653 | 91.04 | 0.83 | 142 | 116 | 3 |
|  | 20 | 2,151 | 2,080 | 96.70 | 0.91 | 28 | 43 | 0 |
|  | 30 | 1,891 | 1,852 | 97.94 | 0.93 | 13 | 26 | 0 |
| M1 | 10 | 3,152 | 2,824 | 89.59 | 0.82 | 137 | 182 | 9 |
|  | 20 | 2,194 | 2,093 | 95.40 | 0.89 | 26 | 74 | 1 |
|  | 30 | 1,831 | 1,778 | 97.11 | 0.91 | 11 | 42 | 0 |
| C1 | 10 | 2,591 | 2141 | 82.63 | 0.74 | 237 | 204 | 9 |
|  | 20 | 920 | 859 | 93.37 | 0.87 | 21 | 40 | 0 |
|  | 30 | 534 | 525 | 98.31 | 0.96 | 2 | 7 | 0 |
| G1 | 10 | 1,688 | 1,451 | 85.96 | 0.73 | 162 | 68 | 7 |
|  | 2 | 429 | 399 | 93.01 | 0.89 | 14 | 16 | 0 |
|  | 30 | 149 | 145 | 97.32 | 0.96 | 2 | 2 | 0 |
| SL1 | 10 | 1,683 | 1,441 | 85.62 | 0.73 | 188 | 52 | 2 |
|  | 20 | 474 | 446 | 94.09 | 0.90 | 14 | 14 | 0 |
|  | 30 | 208 | 201 | 96.63 | 0.94 | 1 | 6 | 0 |
| SL2 | 10 | 2,298 | 1,940 | 84.42 | 0.68 | 244 | 112 | 2 |
|  | 20 | 525 | 481 | 91.62 | 0.86 | 21 | 23 | 0 |
|  | 30 | 234 | 227 | 97.01 | 0.95 | 2 | 5 | 0 |
| U1 | 10 | 1,447 | 1,236 | 85.42 | 0.73 | 144 | 63 | 4 |
|  | 20 | 416 | 381 | 91.59 | 0.87 | 13 | 22 | 0 |
|  | 30 | 184 | 175 | 95.11 | 0.91 | 2 | 7 | 0 |

**Table S3.** Raw sequencing metrics and percentage core genome coverages at coverage depths of 5-fold and 10-fold for sequencing technical repeats of samples.

|  | | **Sample and Timepoint (SWGA Duration)** | | | |
| --- | --- | --- | --- | --- | --- |
|  |  | **IC2b** | | **K1b** | |
|  |  | **8h** | **16h** | **8h** | **16h** |
| **Total number of reads** | | 382,449 | 274,347 | 237,955 | 137,354 |
| **Proportion of reads mapped to *Plasmodium*** | | 47% | 42% | 45% | 38% |
| **Proportion of reads mapped to human** | | 14% | 14% | 13% | 12% |
| **Percentage core genome coverage at ≥5-fold depth** | **Including Apicoplast and Mitochondria Genome** | 62.56% | 49.50% | 53.56% | 28.86% |
|  | **Excluding Apicoplast and Mitochondria Genome** | 70.30% | 56.39% | 58.82% | 32.20% |
| **Percentage core genome coverage at ≥10-fold depth** | **Including Apicoplast and Mitochondria Genome** | 41.79% | 29.59% | 31.07% | 12.62% |
|  | **Excluding Apicoplast and Mitochondria Genome** | 47.61% | 33.81% | 34.93% | 14.39% |

**Table S4. SNP concordance for technical repeats.**

| **Samples from SWGA Incubation Durations** | | | | | **IC2b** | | | **K1b** | | |
| --- | --- | --- | --- | --- | --- | --- | --- | --- | --- | --- |
| **Comparison Group** | | | | | **Original and Repeat 8h Time-point** | **Original and Repeat 16h Time-point** | **Repeat 8h and 16h Time-point** | **Original and Repeat 8h Time-point** | **Original and Repeat 16h Time-point** | **Repeat 8h and 16h Time-point** |
| **Threshold for Determining Allelic Genotype Classification** | **70% Alternate Classification** | **≥10-fold Depth** | **Total SNPs Called** | | 5,921 | 4,245 | 5,575 | 5,223 | 3,589 | 3,194 |
|  |  |  | **Concordant SNPs** | | 5,202 | 3,772 | 4,943 | 4,579 | 2,992 | 2,778 |
|  |  |  | **% Concordance** | | 87.86% | 88.86% | 88.66% | 87.67% | 83.37% | 86.98% |
|  |  |  | **Cohen’s Kappa** | | 0.79 | 0.81 | 0.81 | 0.79 | 0.70 | 0.77 |
|  |  |  | **Discrepancy Type** | **MA** | 379 | 218 | 313 | 270 | 213 | 175 |
|  |  |  |  | **MR** | 265 | 206 | 248 | 301 | 311 | 205 |
|  |  |  |  | **AR** | 75 | 49 | 71 | 73 | 73 | 36 |
|  |  | **≥20-fold Depth** | **Total SNPs Called** | | 4,011 | 3,128 | 3,954 | 3,839 | 2,790 | 2,467 |
|  |  |  | **Concordant SNPs** | | 3,699 | 2,895 | 3,682 | 3,563 | 2,470 | 2,243 |
|  |  |  | **% Concordance** | | 92.22% | 92.55% | 93.12% | 92.81% | 88.53% | 90.92% |
|  |  |  | **Cohen’s Kappa** | | 0.84 | 0.84 | 0.86 | 0.84 | 0.72 | 0.80 |
|  |  |  | **Discrepancy Type** | **MA** | 151 | 90 | 131 | 86 | 63 | 76 |
|  |  |  |  | **MR** | 141 | 132 | 129 | 180 | 219 | 127 |
|  |  |  |  | **AR** | 20 | 11 | 12 | 10 | 38 | 21 |
|  |  | **≥30-fold Depth** | **Total SNPs Called** | | 3,138 | 2,566 | 3,288 | 3,079 | 2,360 | 2,212 |
|  |  |  | **Concordant SNPs** | | 3,013 | 2,464 | 3,132 | 2,945 | 2,159 | 2,044 |
|  |  |  | **% Concordance** | | 96.02% | 96.02% | 95.26% | 95.65% | 91.48% | 92.41% |
|  |  |  | **Cohen’s Kappa** | | 0.89 | 0.89 | 0.88 | 0.87 | 0.75 | 0.81 |
|  |  |  | **Discrepancy Type** | **MA** | 46 | 35 | 78 | 37 | 39 | 48 |
|  |  |  |  | **MR** | 77 | 65 | 71 | 94 | 140 | 109 |
|  |  |  |  | **AR** | 2 | 2 | 7 | 3 | 22 | 11 |
|  | **80% Alternate Classification** | **≥10-fold Depth** | **Total SNPs Called** | | 5,921 | 4,245 | 5,575 | 5,223 | 3,589 | 3,194 |
|  |  |  | **Concordant SNPs** | | 5,225 | 3,745 | 4,921 | 4,580 | 3,026 | 2,767 |
|  |  |  | **% Concordance** | | 88.25% | 88.22% | 88.27% | 87.69% | 84.31% | 86.63% |
|  |  |  | **Cohen’s Kappa** | | 0.81 | 0.80 | 0.81 | 0.79 | 0.71 | 0.76 |
|  |  |  | **Discrepancy Type** | **MA** | 357 | 245 | 335 | 269 | 170 | 186 |
|  |  |  |  | **MR** | 317 | 235 | 292 | 333 | 365 | 229 |
|  |  |  |  | **AR** | 23 | 20 | 27 | 41 | 19 | 12 |
|  |  | **≥20-fold Depth** | **Total SNPs Called** | | 4,011 | 3,128 | 3,954 | 3,839 | 2,790 | 2,467 |
|  |  |  | **Concordant SNPs** | | 3,734 | 2,896 | 3,704 | 3,574 | 2,487 | 2,247 |
|  |  |  | **% Concordance** | | 93.09% | 92.58% | 93.68% | 93.10% | 89.14% | 91.08% |
|  |  |  | **Cohen’s Kappa** | | 0.86 | 0.84 | 0.87 | 0.85 | 0.74 | 0.80 |
|  |  |  | **Discrepancy Type** | **MA** | 116 | 89 | 109 | 75 | 46 | 72 |
|  |  |  |  | **MR** | 157 | 142 | 139 | 188 | 251 | 142 |
|  |  |  |  | **AR** | 4 | 1 | 2 | 2 | 6 | 6 |
|  |  | **≥30-fold Depth** | **Total SNPs Called** | | 3,138 | 2,566 | 3,288 | 3,079 | 2,360 | 2,212 |
|  |  |  | **Concordant SNPs** | | 3,021 | 2,466 | 3,155 | 2,949 | 2,172 | 2,043 |
|  |  |  | **% Concordance** | | 96.27% | 96.10% | 95.95% | 95.78% | 92.03% | 92.36% |
|  |  |  | **Cohen’s Kappa** | | 0.90 | 0.89 | 0.90 | 0.88 | 0.77 | 0.81 |
|  |  |  | **Discrepancy Type** | **MA** | 38 | 33 | 55 | 33 | 26 | 49 |
|  |  |  |  | **MR** | 79 | 66 | 78 | 96 | 160 | 120 |
|  |  |  |  | **AR** | 0 | 1 | 0 | 1 | 2 | 0 |
|  | **90% Alternate Classification** | **≥10-fold Depth** | **Total SNPs Called** | | 5,921 | 4,245 | 5,575 | 5,223 | 3,589 | 3,194 |
|  |  |  | **Concordant SNPs** | | 5,280 | 3,765 | 4,984 | 4,581 | 3,087 | 2,822 |
|  |  |  | **% Concordance** | | 89.17% | 88.69% | 89.40% | 87.70% | 86.01% | 88.35% |
|  |  |  | **Cohen’s Kappa** | | 0.83 | 0.81 | 0.83 | 0.79 | 0.74 | 0.79 |
|  |  |  | **Discrepancy Type** | **MA** | 301 | 225 | 272 | 268 | 118 | 131 |
|  |  |  |  | **MR** | 332 | 245 | 305 | 360 | 380 | 238 |
|  |  |  |  | **AR** | 8 | 10 | 14 | 24 | 4 | 3 |
|  |  | **≥20-fold Depth** | **Total SNPs Called** | | 4,011 | 3,128 | 3,954 | 3,839 | 2,790 | 2,467 |
|  |  |  | **Concordant SNPs** | | 3,794 | 2,928 | 3,751 | 3,590 | 2,507 | 2,294 |
|  |  |  | **% Concordance** | | 94.59% | 93.61% | 94.87% | 93.51% | 89.86% | 92.99% |
|  |  |  | **Cohen’s Kappa** | | 0.89 | 0.86 | 0.90 | 0.85 | 0.75 | 0.84 |
|  |  |  | **Discrepancy Type** | **MA** | 56 | 57 | 62 | 59 | 26 | 25 |
|  |  |  |  | **MR** | 161 | 143 | 141 | 190 | 256 | 148 |
|  |  |  |  | **AR** | 0 | 0 | 0 | 0 | 1 | 0 |
|  |  | **≥30-fold Depth** | **Total SNPs Called** | | 3,138 | 2,566 | 3,288 | 3,079 | 2,360 | 2,212 |
|  |  |  | **Concordant SNPs** | | 3,040 | 2,477 | 3,188 | 2,961 | 2,186 | 2,076 |
|  |  |  | **% Concordance** | | 96.88% | 96.53% | 96.96% | 96.17% | 92.63% | 93.86% |
|  |  |  | **Cohen’s Kappa** | | 0.92 | 0.90 | 0.93 | 0.89 | 0.78 | 0.84 |
|  |  |  | **Discrepancy Type** | **MA** | 19 | 22 | 22 | 21 | 12 | 16 |
|  |  |  |  | **MR** | 79 | 67 | 78 | 97 | 162 | 120 |
|  |  |  |  | **AR** | 0 | 0 | 0 | 0 | 0 | 0 |

**Table S5.** Detailed breakdown of the number of SNPs and concordances between same samples processed by the EquiPhi29™ SWGA protocol and the original protocol using *phi29* (NEB) at incubation durations of 8h and 16h. MA = Mixed and Alternate calls, MR = Mixed and Reference calls, and AR = Alternate and Reference calls.

| **Sample** | | | | | **IC1** | | **C1** | | **M1** | |
| --- | --- | --- | --- | --- | --- | --- | --- | --- | --- | --- |
| **Comparison Group** | | | | | **8h Timepoint** | **16h Timepoint** | **8h Timepoint** | **16h Timepoint** | **8h Timepoint** | **16h Timepoint** |
| **Threshold for Determining Genotype Classification** | **70% Alternative Reads of Total Depth** | **≥10-fold Depth** | **Total SNPs Called** | | 4,898 | 5,676 | 2,919 | 2,802 | 2,647 | 2,927 |
|  |  |  | **Concordant SNPs** | | 3,638 | 4,282 | 2,468 | 2,458 | 1,645 | 1,785 |
|  |  |  | **% Concordance** | | 74.28% | 75.44% | 84.55% | 87.72% | 62.15% | 60.98% |
|  |  |  | **Cohen’s Kappa** | | 0.60 | 0.61 | 0.73 | 0.69 | 0.20 | 0.18 |
|  |  |  | **Discrepancy Type** | **MA** | 342 | 428 | 226 | 207 | 41 | 49 |
|  |  |  |  | **MR** | 719 | 727 | 143 | 90 | 210 | 257 |
|  |  |  |  | **AR** | 199 | 239 | 82 | 47 | 750 | 836 |
|  |  | **≥20-fold Depth** | **Total SNPs Called** | | 1,528 | 1,772 | 616 | 722 | 1,245 | 1,484 |
|  |  |  | **Concordant SNPs** | | 1,248 | 1,481 | 573 | 636 | 956 | 1,126 |
|  |  |  | **% Concordance** | | 81.68% | 83.58% | 93.02% | 88.09% | 76.79% | 75.88% |
|  |  |  | **Cohen’s Kappa** | | 0.67 | 0.70 | 0.85 | 0.80 | 0.30 | 0.22 |
|  |  |  | **Discrepancy Type** | **MA** | 60 | 64 | 21 | 42 | 4 | 11 |
|  |  |  |  | **MR** | 199 | 209 | 16 | 36 | 76 | 113 |
|  |  |  |  | **AR** | 21 | 18 | 6 | 8 | 208 | 234 |
|  |  | **≥30-fold Depth** | **Total SNPs Called** | | 542 | 709 | 248 | 271 | 922 | 1,021 |
|  |  |  | **Concordant SNPs** | | 496 | 646 | 244 | 251 | 781 | 863 |
|  |  |  | **% Concordance** | | 91.51% | 91.11% | 98.39% | 92.62% | 84.70% | 84.52% |
|  |  |  | **Cohen’s Kappa** | | 0.79 | 0.78 | 0.95 | 0.86 | 0.33 | 0.29 |
|  |  |  | **Discrepancy Type** | **MA** | 12 | 15 | 2 | 10 | 1 | 5 |
|  |  |  |  | **MR** | 32 | 44 | 2 | 9 | 44 | 40 |
|  |  |  |  | **AR** | 2 | 4 | 0 | 1 | 95 | 113 |
|  | **80% Alternative Reads of Total Depth** | **≥10-fold Depth** | **Total SNPs Called** | | 4,898 | 5,676 | 2,919 | 2,802 | 2,647 | 2,927 |
|  |  |  | **Concordant SNPs** | | 3,627 | 4,291 | 2,327 | 2,313 | 1,629 | 1,778 |
|  |  |  | **% Concordance** | | 74.05% | 75.60% | 79.72% | 82.55% | 61.54% | 60.74% |
|  |  |  | **Cohen’s Kappa** | | 0.61 | 0.63 | 0.67 | 0.64 | 0.21 | 0.19 |
|  |  |  | **Discrepancy Type** | **MA** | 353 | 419 | 367 | 352 | 57 | 56 |
|  |  |  |  | **MR** | 849 | 877 | 182 | 104 | 310 | 353 |
|  |  |  |  | **AR** | 69 | 89 | 43 | 33 | 650 | 740 |
|  |  | **≥20-fold Depth** | **Total SNPs Called** | | 1,528 | 1,772 | 616 | 722 | 1,245 | 1,484 |
|  |  |  | **Concordant SNPs** | | 1,262 | 1,500 | 553 | 601 | 950 | 1,124 |
|  |  |  | **% Concordance** | | 82.59% | 84.65% | 89.77% | 83.24% | 76.31% | 75.74% |
|  |  |  | **Cohen’s Kappa** | | 0.69 | 0.72 | 0.79 | 0.73 | 0.29 | 0.22 |
|  |  |  | **Discrepancy Type** | **MA** | 46 | 45 | 41 | 77 | 10 | 13 |
|  |  |  |  | **MR** | 214 | 221 | 20 | 38 | 107 | 147 |
|  |  |  |  | **AR** | 6 | 6 | 2 | 6 | 177 | 200 |
|  |  | **≥30-fold Depth** | **Total SNPs Called** | | 542 | 709 | 248 | 271 | 922 | 1,021 |
|  |  |  | **Concordant SNPs** | | 500 | 652 | 235 | 241 | 778 | 861 |
|  |  |  | **% Concordance** | | 92.25% | 91.96% | 94.76% | 88.93% | 84.38% | 84.32% |
|  |  |  | **Cohen’s Kappa** | | 0.81 | 0.80 | 0.82 | 0.80 | 0.32 | 0.29 |
|  |  |  | **Discrepancy Type** | **MA** | 8 | 9 | 11 | 20 | 4 | 7 |
|  |  |  |  | **MR** | 33 | 47 | 2 | 10 | 55 | 59 |
|  |  |  |  | **AR** | 1 | 1 | 0 | 0 | 84 | 94 |
|  | **90% Alternative Reads of Total Depth** | **≥10-fold Depth** | **Total SNPs Called** | | 4,898 | 5,676 | 2,919 | 2,802 | 2,647 | 2,927 |
|  |  |  | **Concordant SNPs** | | 3,675 | 4,377 | 2,141 | 2,057 | 1,611 | 1,770 |
|  |  |  | **% Concordance** | | 75.03% | 77.11% | 73.35% | 73.41% | 60.86% | 60.47% |
|  |  |  | **Cohen’s Kappa** | | 0.62 | 0.65 | 0.60 | 0.55 | 0.21 | 0.20 |
|  |  |  | **Discrepancy Type** | **MA** | 305 | 333 | 553 | 608 | 75 | 64 |
|  |  |  |  | **MR** | 896 | 934 | 208 | 116 | 438 | 510 |
|  |  |  |  | **AR** | 22 | 32 | 17 | 21 | 522 | 583 |
|  |  | **≥20-fold Depth** | **Total SNPs Called** | | 1,528 | 1,772 | 616 | 722 | 1,245 | 1,484 |
|  |  |  | **Concordant SNPs** | | 1,281 | 1,513 | 536 | 563 | 947 | 1,127 |
|  |  |  | **% Concordance** | | 83.84% | 85.38% | 87.01% | 77.98% | 76.06% | 75.94% |
|  |  |  | **Cohen’s Kappa** | | 0.71 | 0.73 | 0.74 | 0.67 | 0.29 | 0.23 |
|  |  |  | **Discrepancy Type** | **MA** | 27 | 32 | 58 | 115 | 13 | 10 |
|  |  |  |  | **MR** | 220 | 226 | 21 | 43 | 147 | 182 |
|  |  |  |  | **AR** | 0 | 1 | 1 | 1 | 137 | 165 |
|  |  | **≥30-fold Depth** | **Total SNPs Called** | | 542 | 709 | 248 | 271 | 922 | 1,021 |
|  |  |  | **Concordant SNPs** | | 501 | 652 | 231 | 234 | 778 | 863 |
|  |  |  | **% Concordance** | | 92.44% | 91.96% | 93.15% | 86.35% | 84.38% | 84.52% |
|  |  |  | **Cohen’s Kappa** | | 0.81 | 0.80 | 0.77 | 0.76 | 0.32 | 0.30 |
|  |  |  | **Discrepancy Type** | **MA** | 7 | 9 | 15 | 27 | 4 | 5 |
|  |  |  |  | **MR** | 34 | 48 | 2 | 10 | 74 | 76 |
|  |  |  |  | **AR** | 0 | 0 | 0 | 0 | 65 | 77 |

**Table S6.** Percentage core genome coverage at 5-fold and 10-fold depths for sequencing technical replicates of samples processed with the EquiPhi29™ SWGA protocol. Because the original samples had more reads than the replicates, their read counts were normalized to match those of the corresponding replicates. For this analysis, the apicoplast and mitochondrial genomes were excluded from the core genome coverage calculations.

|  | | **Sample and Timepoint (SWGA Duration)** | | |
| --- | --- | --- | --- | --- |
|  |  | **IC1b** | **C1b** | **M1b** |
|  |  |  |  |  |
| **Percentage Core Genome Coverage at ≥5-fold Depth** | **Including Apicoplast and Mitochondria Genome** | 71.79% | 66.19% | 57.98% |
|  | **Excluding Apicoplast and Mitochondria Genome** | 74.93% | 68.40% | 64.36% |
| **Percentage Core Genome Coverage at ≥10-fold Depth** | **Including Apicoplast and Mitochondria Genome** | 46.52% | 37.58% | 39.49% |
|  | **Excluding Apicoplast and Mitochondria Genome** | 46.07% | 35.84% | 44.75% |

**Table S7.** Individual SNP concordance of all technical repeats for all samples using the new Equiphi29™ polymerase. MA = Mixed and Alternate calls, MR = Mixed and Reference calls, and AR = Alternate and Reference calls.

| **Samples from SWGA Incubation Durations** | | | | | **IC1b** | | | **C1b** | | | **M1b** | | |
| --- | --- | --- | --- | --- | --- | --- | --- | --- | --- | --- | --- | --- | --- |
| **Comparison Group** | | | | | **IC1 Original** | **8h Time-point** | **16h Time-point** | **C1 Original** | **8h Time-point** | **16h Time-point** | **M1 Original** | **8h Time-point** | **16h Time-point** |
| **Threshold for Determining Allelic Genotype Classification** | **70% Alternative Reads of Total Depth** | **≥10-fold Depth** | **Total SNPs Called** | | 13,109 | 4,898 | 4,565 | 8,626 | 2,919 | 2,620 | 17,951 | 2,647 | 4,312 |
|  |  |  | **Concordant SNPs** | | 10,853 | 3,638 | 3,399 | 7,860 | 2,468 | 2,283 | 17,250 | 1,645 | 2,912 |
|  |  |  | **% Concordance** | | 82.79% | 74.28% | 74.46% | 91.11% | 84.55% | 87.14% | 96.10% | 62.15% | 67.53% |
|  |  |  | **Cohen’s Kappa** | | 0.68 | 0.60 | 0.60 | 0.84 | 0.73 | 0.72 | 0.92 | 0.20 | 0.24 |
|  |  |  | **Discrepancy Type** | **MA** | 592 | 342 | 352 | 310 | 226 | 191 | 311 | 41 | 48 |
|  |  |  |  | **MR** | 1,384 | 719 | 608 | 363 | 143 | 91 | 334 | 210 | 280 |
|  |  |  |  | **AR** | 280 | 199 | 206 | 93 | 82 | 55 | 56 | 750 | 1,072 |
|  |  | **≥20-fold Depth** | **Total SNPs Called** | | 5,960 | 1,528 | 1,274 | 3,068 | 616 | 748 | 14,288 | 1,245 | 2,195 |
|  |  |  | **Concordant SNPs** | | 5,413 | 1,248 | 1,058 | 2,966 | 573 | 688 | 14,056 | 956 | 1,649 |
|  |  |  | **% Concordance** | | 90.82% | 81.68% | 83.05% | 96.68% | 93.02% | 91.98% | 98.38% | 76.79% | 75.13% |
|  |  |  | **Cohen’s Kappa** | | 0.76 | 0.67 | 0.68 | 0.93 | 0.85 | 0.87 | 0.96 | 0.30 | 0.22 |
|  |  |  | **Discrepancy Type** | **MA** | 139 | 60 | 45 | 40 | 21 | 32 | 105 | 4 | 18 |
|  |  |  |  | **MR** | 382 | 199 | 160 | 59 | 16 | 26 | 120 | 76 | 156 |
|  |  |  |  | **AR** | 26 | 21 | 11 | 3 | 6 | 2 | 7 | 208 | 372 |
|  |  | **≥30-fold Depth** | **Total SNPs Called** | | 3,145 | 542 | 489 | 1,604 | 248 | 377 | 12,308 | 922 | 1,488 |
|  |  |  | **Concordant SNPs** | | 2,990 | 496 | 446 | 1,579 | 244 | 368 | 12,215 | 781 | 1,232 |
|  |  |  | **% Concordance** | | 95.07% | 91.51% | 91.21% | 98.44% | 98.39% | 97.61% | 99.24% | 84.71% | 82.80% |
|  |  |  | **Cohen’s Kappa** | | 0.82 | 0.79 | 0.77 | 0.94 | 0.95 | 0.95 | 0.97 | 0.33 | 0.30 |
|  |  |  | **Discrepancy Type** | **MA** | 36 | 12 | 9 | 4 | 2 | 6 | 37 | 1 | 11 |
|  |  |  |  | **MR** | 118 | 32 | 31 | 19 | 2 | 3 | 55 | 44 | 78 |
|  |  |  |  | **AR** | 1 | 2 | 3 | 2 | 0 | 0 | 1 | 95 | 167 |
|  | **80% Alternative Reads of Total Depth** | **≥10-fold Depth** | **Total SNPs Called** | | 13,109 | 4,898 | 4,565 | 8,626 | 2,919 | 2,620 | 17,951 | 2,647 | 4,312 |
|  |  |  | **Concordant SNPs** | | 10,937 | 3,627 | 3,432 | 7,840 | 2,327 | 2,163 | 17,169 | 1,629 | 2,900 |
|  |  |  | **% Concordance** | | 83.43% | 74.05% | 75.18% | 90.89% | 79.72% | 82.56% | 95.64% | 61.54% | 67.25% |
|  |  |  | **Cohen’s Kappa** | | 0.70 | 0.61 | 0.62 | 0.85 | 0.67 | 0.67 | 0.91 | 0.21 | 0.25 |
|  |  |  | **Discrepancy Type** | **MA** | 508 | 353 | 319 | 330 | 367 | 311 | 392 | 57 | 60 |
|  |  |  |  | **MR** | 1,558 | 849 | 735 | 428 | 182 | 120 | 369 | 310 | 410 |
|  |  |  |  | **AR** | 106 | 69 | 79 | 28 | 43 | 26 | 21 | 650 | 942 |
|  |  | **≥20-fold Depth** | **Total SNPs Called** | | 5,960 | 1,528 | 1,274 | 3,068 | 616 | 748 | 14,288 | 1,245 | 2,195 |
|  |  |  | **Concordant SNPs** | | 5,445 | 1,262 | 1,074 | 2,966 | 553 | 654 | 14,016 | 950 | 1,645 |
|  |  |  | **% Concordance** | | 91.36% | 82.59% | 84.30% | 96.68% | 89.77% | 87.43% | 98.10% | 76.31% | 74.94% |
|  |  |  | **Cohen’s Kappa** | | 0.77 | 0.69 | 0.71 | 0.93 | 0.79 | 0.80 | 0.95 | 0.29 | 0.22 |
|  |  |  | **Discrepancy Type** | **MA** | 107 | 46 | 29 | 40 | 41 | 66 | 145 | 10 | 22 |
|  |  |  |  | **MR** | 400 | 214 | 170 | 62 | 20 | 28 | 126 | 107 | 222 |
|  |  |  |  | **AR** | 8 | 6 | 1 | 0 | 2 | 0 | 1 | 177 | 306 |
|  |  | **≥30-fold Depth** | **Total SNPs Called** | | 3,145 | 542 | 489 | 1,604 | 248 | 377 | 12,308 | 922 | 1,488 |
|  |  |  | **Concordant SNPs** | | 2,999 | 500 | 452 | 1,575 | 235 | 355 | 12,197 | 778 | 1,229 |
|  |  |  | **% Concordance** | | 95.36% | 92.25% | 92.43% | 98.19% | 94.76% | 94.16% | 99.10% | 84.38% | 82.59% |
|  |  |  | **Cohen Kappa** | | 0.83 | 0.81 | 0.80 | 0.93 | 0.82 | 0.88 | 0.97 | 0.32 | 0.29 |
|  |  |  | **Discrepancy Type** | **MA** | 27 | 8 | 3 | 8 | 11 | 19 | 55 | 4 | 14 |
|  |  |  |  | **MR** | 118 | 33 | 34 | 21 | 2 | 3 | 56 | 55 | 104 |
|  |  |  |  | **AR** | 1 | 1 | 0 | 0 | 0 | 0 | 0 | 84 | 141 |
|  | **90% Alternative Reads of Total Depth** | **≥10-fold Depth** | **Total SNPs Called** | | 13,109 | 4,898 | 4,565 | 8,626 | 2,919 | 2,620 | 17,951 | 2,647 | 4,312 |
|  |  |  | **Concordant SNPs** | | 11,037 | 3,675 | 3,464 | 7,766 | 2,141 | 1,988 | 17,101 | 1,611 | 2,884 |
|  |  |  | **% Concordance** | | 84.19% | 75.03% | 75.88% | 90.03% | 73.35% | 75.88% | 95.26% | 60.86% | 66.88% |
|  |  |  | **Cohen Kappa** | | 0.71 | 0.62 | 0.63 | 0.84 | 0.60 | 0.61 | 0.91 | 0.21 | 0.25 |
|  |  |  | **Discrepancy Type** | **MA** | 408 | 305 | 287 | 404 | 553 | 486 | 460 | 75 | 76 |
|  |  |  |  | **MR** | 1,628 | 896 | 783 | 453 | 208 | 136 | 384 | 438 | 616 |
|  |  |  |  | **AR** | 36 | 22 | 31 | 3 | 17 | 10 | 6 | 522 | 736 |
|  |  | **≥20-fold Depth** | **Total SNPs Called** | | 5,960 | 1,528 | 1,274 | 3,068 | 616 | 748 | 14,288 | 1,245 | 2,195 |
|  |  |  | **Concordant SNPs** | | 5,494 | 1,281 | 1,079 | 2,964 | 536 | 620 | 14,016 | 947 | 1,647 |
|  |  |  | **% Concordance** | | 92.18% | 83.84% | 84.69% | 96.61% | 87.01% | 82.89% | 98.10% | 76.06% | 75.03% |
|  |  |  | **Cohen’s Kappa** | | 0.79 | 0.71 | 0.71 | 0.93 | 0.74 | 0.74 | 0.95 | 0.29 | 0.23 |
|  |  |  | **Discrepancy Type** | **MA** | 58 | 27 | 24 | 42 | 58 | 100 | 124 | 13 | 20 |
|  |  |  |  | **MR** | 408 | 220 | 171 | 62 | 21 | 28 | 127 | 147 | 299 |
|  |  |  |  | **AR** | 0 | 0 | 0 | 0 | 1 | 0 | 0 | 137 | 229 |
|  |  | **≥30x Depth** | **Total SNPs Called** | | 3,145 | 542 | 489 | 1,604 | 248 | 377 | 12,308 | 922 | 1,488 |
|  |  |  | **Concordant SNPs** | | 3,016 | 501 | 452 | 1,578 | 231 | 347 | 12,215 | 778 | 1,234 |
|  |  |  | **% Concordance** | | 95.90% | 92.44% | 92.43% | 98.38% | 93.15% | 92.04% | 99.24% | 84.38% | 82.93% |
|  |  |  | **Cohen’s Kappa** | | 0.85 | 0.81 | 0.80 | 0.94 | 0.77 | 0.84 | 0.97 | 0.32 | 0.30 |
|  |  |  | **Discrepancy Type** | **MA** | 10 | 7 | 3 | 5 | 15 | 27 | 37 | 4 | 9 |
|  |  |  |  | **MR** | 119 | 34 | 34 | 21 | 2 | 3 | 36 | 74 | 144 |
|  |  |  |  | **AR** | 0 | 0 | 0 | 0 | 0 | 0 | 0 | 65 | 101 |

**Figure S1**. Flowchart to demonstrate the use of samples and conditions.


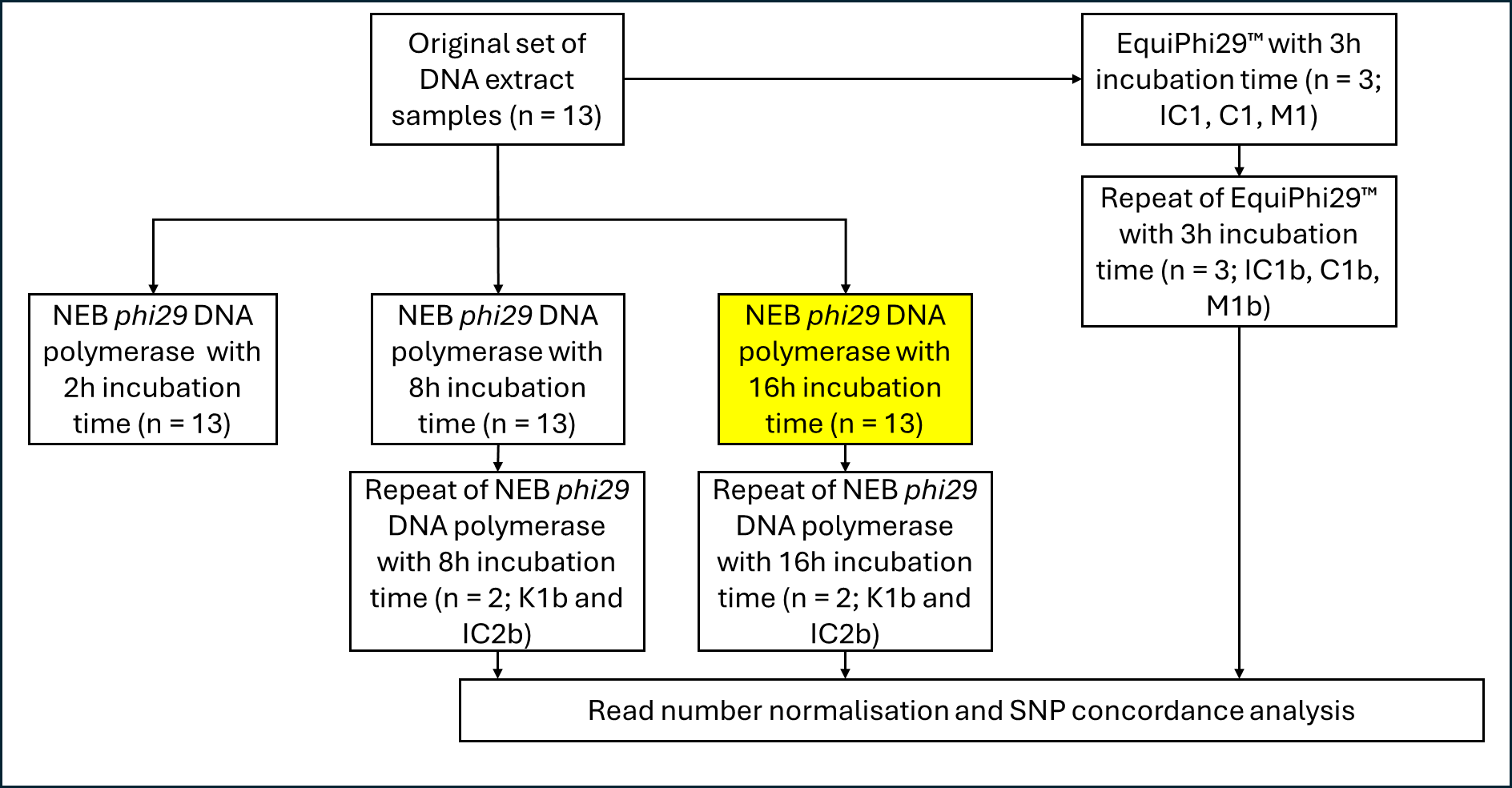


| **Figure S2.** The mean percentage genome coverage of each sample ran on the different protocols, categorized by the chromosomes from the reference genome. P29 refers to the current *phi29* DNA polymerase in use, which means samples ran on the current protocol, and EQ29 refers to EquiPhi29™, which means samples ran on the second tested SWGA protocol. |
| --- |


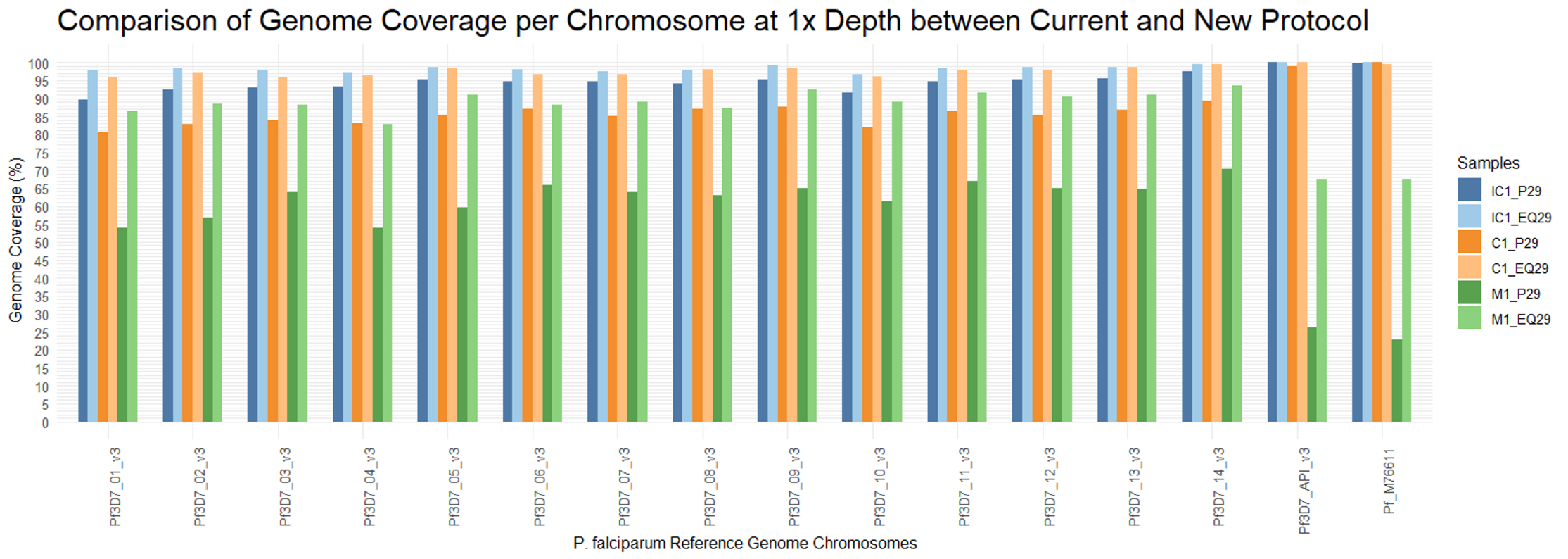

Supplement: Supplementary file 1 — Supplementary material 1. [file 12936_2025_5643_MOESM1_ESM.docx]
